# Randomized single-blind study to examine the implementation and effectiveness of integrating evidence-based early relational health programs in pediatrics for families with low incomes: A clinical trial protocol

**DOI:** 10.1371/journal.pone.0354239

**Published:** 2026-07-24

**Authors:** Caitlin F. Canfield, Elizabeth B. Miller

**Affiliations:** 1 Department of Pediatrics, NYU Grossman School of Medicine, New York, New York, United States of America; 2 Department of Population Health, NYU Grossman School of Medicine, New York, New York, United States of America; PLOS: Public Library of Science, UNITED KINGDOM OF GREAT BRITAIN AND NORTHERN IRELAND

## Abstract

Disparities in early childhood development due to poverty are linked to lifelong gaps in health and well-being. To address such disparities, interventions must not only include the prevention of toxic stress, but also the promotion of early relational health—that is the presence of safe, stable, nurturing relationships between young children and their caregivers. Moreover, a public health approach that integrates multiple programs in order to meet the unique needs and build on the varied resources of individual families is needed to effect population-level change. This randomized controlled trial seeks to examine the implementation and effectiveness of combining two evidence-based interventions, HealthySteps and PlayReadVIP. A total of 355 parent-child dyads, 35 in the implementation phase and 320 in the effectiveness phase, will be randomly assigned to receive HealthySteps+PlayReadVIP or traditional HealthySteps, as an active Control. Families will receive the intervention at each pediatric well-child visit from birth to age 2 and will complete assessments at enrollment and child age 12 and 24 months. In addition, up to ten HealthySteps Specialists and other care providers will be enrolled and will complete surveys at each assessment point. The primary outcomes include positive parent-child relationship, parent cognitive stimulation, and child socioemotional development.

## Introduction

Poverty is linked to disparities in early child development (ECD), a core contributor to long-term physical and mental health [1,2]. These disparities begin early in life [3,4], widen as children age [1], persist across the lifespan [1,2], and stem from broad contextual factors like social determinants of health (SDOH) resulting in inequality of resources and opportunity.

**Fig 1:**
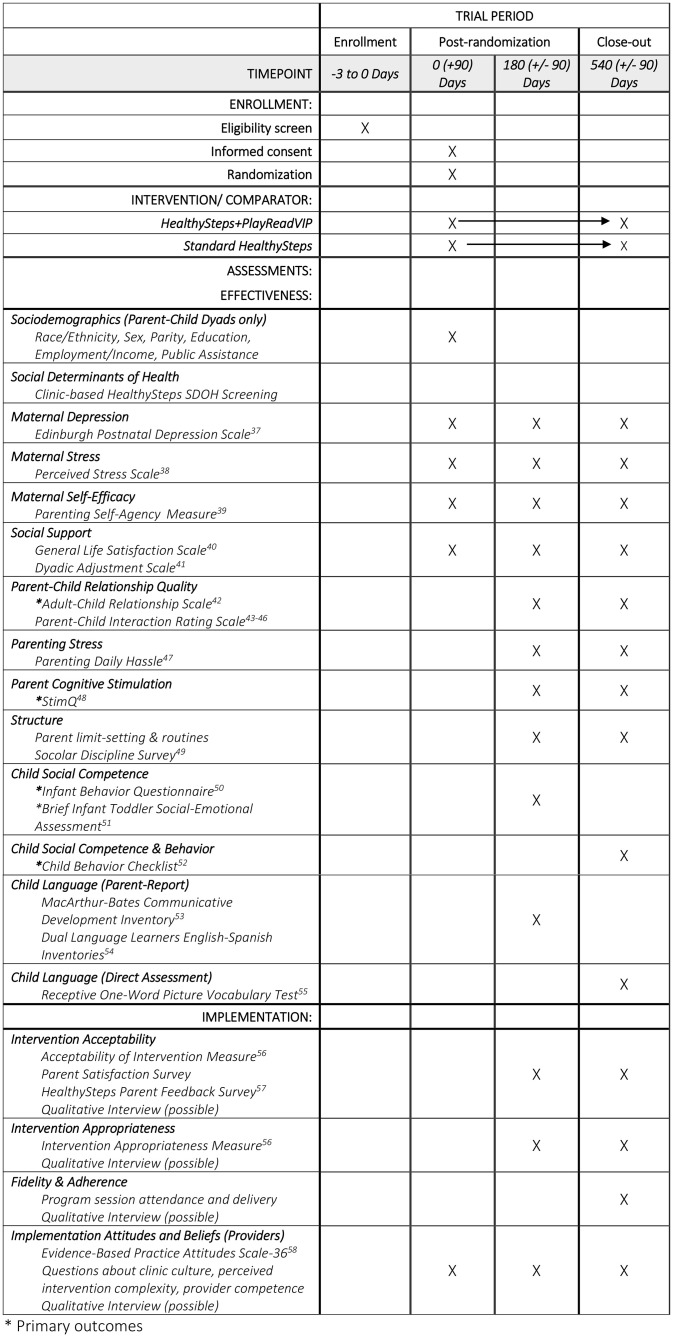
Participant timeline: Schedule of enrollment, interventions, and assessments.

As pediatric primary care (PPC) providers confront these pervasive disparities in ECD, there has been increasing awareness that preventing childhood toxic stress [5] must also include a focus on Early Relational Health (ERH), or the presence of safe, stable, nurturing relationships between children and their caregivers [6]. Acknowledging the many and varied needs of families in order to promote ERH, the American Academy of Pediatrics (AAP) [6] and the National Academies of Sciences, Engineering, and Medicine (NASEM) [7,8] call for the integration and coordination of multiple ERH programs in order the best serve families in meeting these needs and benefitting the parent-child relationship. Here we describe the study protocol of a randomized controlled trial (RCT) aimed at examining such integrations (Fig 1).

**Fig 2:**
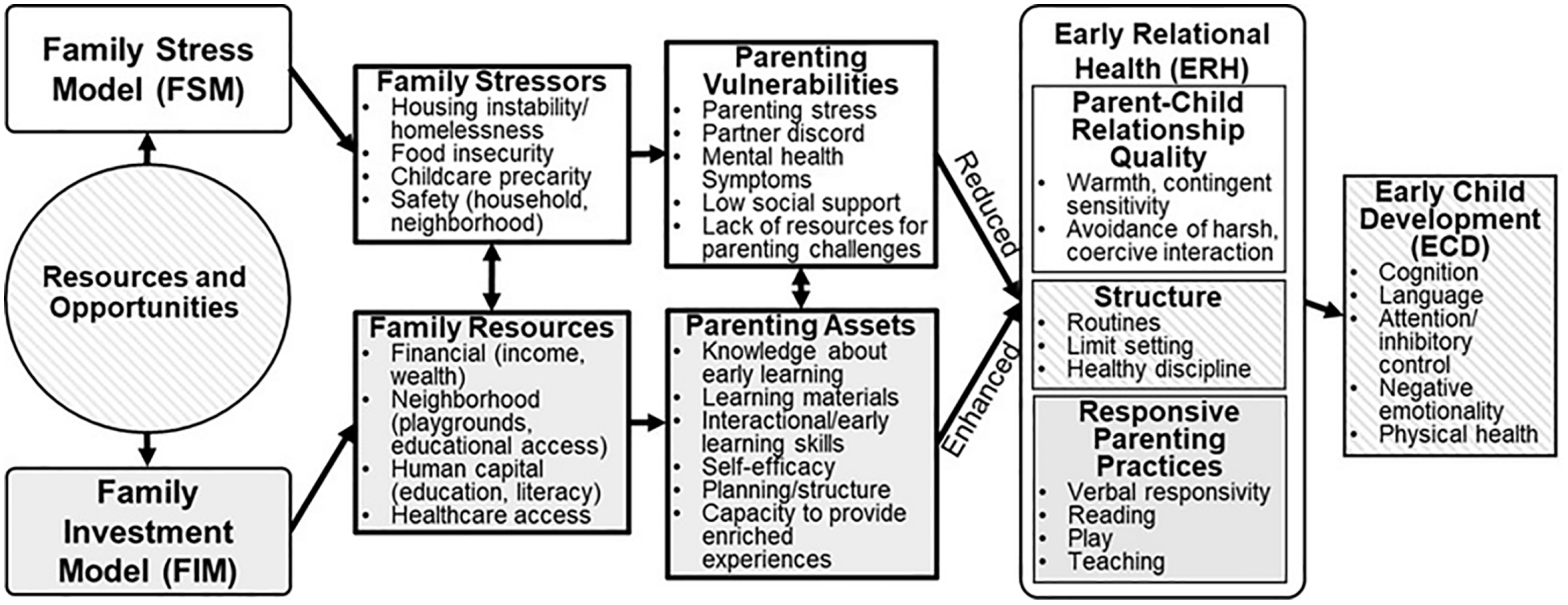
The family stress and investment models.

ERH can be conceptualized as a convergent outcome within two interrelated frameworks with broad theoretical and empirical support (family stress and family investment models; Fig 2), reinforcing its salience as a target for early preventive intervention [9]. The family stress model [10] posits that inequality of resources and opportunity impact ECD [11] through parent emotional/relational distress [12] and consequent effects on relationship quality components of ERH (e.g., increased harsh interactions). The family investment model [13] posits that inequality of resources and opportunity impact ECD by limiting resources and parent assets, particularly time and money, thereby limiting capacity for provision of learning materials (books, toys), and reducing prospects for one-on-one interactions that promote children’s learning skills, with consequent reduction in the responsivity/cognitive stimulation components of ERH (e.g., reading, teaching, and play [14–17]).

Programs that promote ERH to benefit the parent-child relationship and that are based in PPC generally fall into three categories [9]: 1) maternal mental health – addressing the mental health needs of the parent in order to reduce barriers to responsive parenting; 2) SDOH – addressing circumstances that make parenting more challenging such as housing and food security; and 3) parenting – promoting responsive parenting practices and supporting parenting self-efficacy. Thus, through either primary or secondary prevention and concomitant impacts on the parent-child relationship, ERH programs can prevent maladaptive activation of the stress response system, thereby preventing toxic stress, and promote the skills that parents and children need to narrow disparities in ECD [2]. Importantly, better ERH is associated with a range of positive outcomes in multiple domains over the lifespan, including socioemotional development, mental health, relationships with family, friends, and romantic partners, as well as physical health [6], again supporting its salience as a target for early preventive intervention.

We propose a novel integration of two exemplary, AAP-recommended ERH programs delivered in PPC. We briefly mention them here and describe them more fully below in Materials and Methods.

1) HealthySteps (HS) utilizes a stepped-care approach including universal screening for maternal mental health/SDOH and child developmental delays. Families with increased concerns also receive customized support and referral for additional services. Studies of HealthySteps, including a large-scale national study that involved randomized and quasi-experimental sites, suggest that families in the program are more likely to maintain their child’s well-child care, including completion of developmental screenings, on-time vaccinations, increased attendance at pediatric visits, and continuing care at their pediatric practice [18–20]. Moreover, parents report increased information about community resources and referrals for services [19], and increased discussions of mental health with their child’s clinician [21]. However, HS does not provide direct services to address parenting, suggesting the need for additional strategies to improve effectiveness in improving ERH.
2) PlayReadVIP has the potential to address this gap as the program video-records the parent and child playing with a toy or reading a book with immediate, real-time review and feedback to identify and reinforce strengths in the interaction. Three RCTs of PlayReadVIP have supported the program’s beneficial impacts on parenting assets (e.g., learning materials [22]), cognitive stimulation (e.g., reading aloud [22,23]), and child outcomes [24], with additional impacts on parent-child relationship quality (e.g., harsh discipline [25]) and structure (e.g., screentime [26]).

The overarching objective of this study is to understand both the effectiveness and practical implementation of an HS+PlayReadVIP integrated model in real-world PPC clinics. Currently, implementing ERH programs like HS and PlayReadVIP separately in PPC clinics presents several challenges such as time limitations, limited funding, space and staffing issues, lack of training, and not reaching the intended patient population. By combining these two exemplary programs, this study will demonstrate the value of integrating evidence-based programs in PPC to address disparities in ECD and generate actionable knowledge about how to implement and sustain such integrated models effectively in healthcare settings.

## Materials and methods

### Aims and study design

This study will utilize an effective-implementation hybrid Type I design to address two aims:

1. Assess the effectiveness of the HS + VIP model on ERH, parent mental health, parenting behaviors, and child outcomes compared to standard HealthySteps (HS).
  - Examine direct impacts of HS+PlayReadVIP on ERH, parent mental health, parenting behaviors, and child outcomes compared to standard HS.
  - Examine whether HS+PlayReadVIP effectiveness for child outcomes is mediated through impacts on ERH, parent mental health, and parenting.
2. Evaluate the implementation outcomes of the HS+PlayReadVIP model.
  - Examine parent and provider perspectives on feasibility, acceptability, and appropriateness, as well as indicators of reach, adherence, and fidelity.
  - Examine whether HS+PlayReadVIP effectiveness is moderated by implementation factors.

The study will incorporate an RCT of 320 families randomized to either HS+PlayReadVIP or standard HS (described below) as an active control for the effectiveness phase, as well as 35 families and up to 10 HS Specialists and other care providers enrolled in the implementation phase of the study. The SPIRIT Participant Timeline (Fig 1) provides a timeline of our enrollment, intervention delivery, and study assessments. The clinical trial protocol approved by the NYUGSOM IRB prior to allocation of participants and data collection is included as S2 Appendix.

### Setting and participants

Enrollment, randomization, and intervention delivery for parent and child participants in both phases of the study will take place at the NYU Langone Sunset Park Family Health Centers (SPFHC), a network of federally-qualified health centers in Sunset Park, Brooklyn. In total from July 2025-April 2026, the SPFHC served 4,189 children ages 0–3 (80% Hispanic, 8% Black/African-American, 3% Asian), and 90% of these families had insurance coverage through Medicaid or CHIP. SDOH screenings during that period revealed that families reported the greatest needs related to utilities (52%), food insecurity (28%), housing (27%), and transportation (13%). Given the large number of families served at the SPFHC, we do not anticipate the need for specific strategies to achieve our enrollment goal. However, if such strategies are needed, we will work with clinic staff and leadership to identify methods for promoting study awareness and participation.

Parent-infant dyads presenting to the FHC pediatric clinics are assessed for eligibility by trained research staff and offered enrollment in the study if they met the following criteria: 1) parent is the child’s legal guardian and at least 18 years of age, 2) Infants are < 6 months of age, 3) parent/guardian speaks English or Spanish, 4) family is identified as needing additional services (i.e., HealthySteps Tier 3, below), 5) parent is able and willing to provide consent for their own and their child’s participation, 6) child is a singleton, 7) parent plans to continue pediatric care at the SPFHC, and 8) parent has no known severe medical or psychiatric impairment that would interfere with study participation.

After confirmation of eligibility, potential participants are provided with information about the study interventions, study procedures, and risks. Informed consent forms approved by the NYUGSOM IRB, detailing all study information, are provided to participants, and participants have the opportunity to review consent materials and ask questions. Before participating in the study, parents must provide written consent for themselves and for their children, either at the time of the consent discussion or after discussing the study with family members and others. Participants are provided with a copy of the consent form and are reminded that they may withdraw consent at any time during the course of the study and that declining or withdrawing consent will not interfere with the quality of their medical care at the SPFHC.

Recruitment for the effectiveness phase of the study will end when a sample of 320 parent-infant dyads have been enrolled in the study. The sample size of 320 provides 80% power to detect an effect of approximately .28 for all analyses in the effectiveness phase.

Recruitment of parent participants for the implementation phase of the study will end when 35 parent-infant dyads have been enrolled. In addition, program and other care providers will be approached through passive recruitment methods to participate in the implementation phase. All HS and PlayReadVIP program providers at the SPFHC (n = 5) along with pediatric clinicians and clinic management (n = 5) will be invited to enroll.

### Randomization and blinding

After enrollment, parent participants in both phases of the study will be randomized to either the HS+PlayReadVIP or standard HS groups using a simple randomization procedure with a maximum tolerated imbalance generated by the study PIs. Random allocation will be implemented through sequential numbers in an electronic database in REDCap, only accessed by a single member of the study team at the time of randomization. Enrollment and randomization will be conducted separately from participant assessments, and research staff conducting assessments will be masked to participant randomization group.

### Interventions

#### HealthySteps (HS).

HealthySteps serves as the active control intervention in this study. HS capitalizes on healthcare redesign efforts using systems-level changes to improve the quality of PPC, particularly for children from households with low incomes [27]. The program combines a tiered model based on risk stratification with integrated and coordinated services for behavioral and mental health, and referral to governmental and community resources to address SDOH. HealthySteps implements universal screening and provides consultation and services, including care coordination, positive parenting guidance, support for parent mental health and child behavior for families in need of additional services (i.e., Tier 2 and Tier 3). In addition, HealthySteps specialists, generally Master’s-level professionals or higher, join team-based well-child visits for families most at risk (i.e., Tier 3).

HS has programs in more than 350 sites in 25 states, Washington DC, and internationally. [28] The HS National Office (HS NO; within Zero to Three [28]) developed an implementation guide to promote consistent delivery, fidelity metrics, performance indicators, and a continuous quality improvement plan across the HS network. Each HS site collaborates with the HS NO to create an Implementation Plan to support 12 delivery fidelity indicators associated with 8 core components, ensuring accountability and tracking progress over time.

#### Integrated HealthySteps and PlayReadVIP (HS+PlayReadVIP).

The intervention of interest in the current study is an integrated model of HealthySteps and PlayReadVIP. This service model adds core components of PlayReadVIP (described below) within the one-on-one sessions provided by the HS Specialist to families identified as Tier 3. The integrated delivery model is purposely flexible, so that the HS Specialist can provide the PlayReadVIP component at any time during the session, depending on the family’s other needs.

#### PlayReadVIP.

PlayReadVIP is a co-located program focused on promoting early relational health (ERH), which is centered around a core component of video-recording and review. At a standalone PlayReadVIP session, families participate in a one-on-one session with a Bachelor’s level “parent coach” who records the parent and child playing or reading for 3 minutes, and then reviews the video with the parent, supporting self-reflection, reinforcing strengths, and highlighting ways to increase responsivity. [29] Sessions take ~20−25 minutes and also include written parent guides to support families at home and provision of developmentally-appropriate toys and/or books, to further enhance parent-child interactions. Sessions are delivered 1-on-1 at the time of well-child visits from birth to age 3. PlayReadVIP coaches complete a 3-day training course, including detailed review of background material, instruction regarding modeling, and culturally-sensitive practice. PlayReadVIP’s low cost and strong evidence base have resulted in increased implementation in PPC, with 25 sites in 6 cities. [30]

For the integrated model, an ~ 10-minute PlayReadVIP component that includes provision of the toy and/or book, 3–5 minute video recording, and review with the parent is added to the HS session. HS Specialists complete the full PlayReadVIP training, with additional training, supervision, and feedback on the integration of the component into the HS session.

### Data collection

Fig 1 provides a list of all variables along with the timing of data collection. These include primary and secondary outcome variables, as well as data unrelated to outcomes, including sociodemographics, as well as pre-intervention (baseline) psychosocial stressors, social determinants of health, social support, and parenting self-efficacy. Data for all variables will be collected through structured interviews with enrolled parent and/or provider participants using validated instruments. In addition, parent-child observations and direct assessments of children’s abilities will be conducted as required by specific measures. All assessments will be conducted by trained research staff who are fluent in both English and Spanish. To promote participant retention, parent-child dyads will be provided with $25 for each parent survey, and $35 for each in-person assessment, including both the parent-child observation and direct child assessment, as compensation for their time and travel.

### Data management and safety

All data will be collected directly in REDCap databases and will be periodically downloaded and backed-up on NYU secure network drives. All video recordings of parent-child interactions and direct child assessments will similarly be stored on NYU network drives. All scores and coding from these assessments will be entered directly into REDCap.

To ensure participant safety and data security, the project will establish a Data and Safety Monitoring Plan in which unanticipated problems or adverse events, if identified, will be documented through a centralized database system, investigated, followed up under the direction of the Principal Investigators, and reported to the IRB.

It is expected that if unanticipated problems or adverse events occur, they will be identified by either: a) families or others communicating directly to the MPIs (whose names and telephone numbers are included on the consent form copy provided to the family); b) communication to the NYUGSOM IRB (similarly the consent form includes the IRB contact number), or c) project staff who learn of a situation through their ongoing contacts with families or health providers. Irrespective of the source, all such occurrences will be both communicated to the MPIs and documented in the project’s computer database, in which an “Adverse Event” field will be available for each subject. This will allow systematic and timely collection of information on possible problems and provide the MPIs the means to monitor their occurrence, investigate and follow up, and document outcomes. Any significant unanticipated problem or adverse events will be immediately reported to the NYU IRB. The results of the MPIs’ investigation of any adverse events or unanticipated problems will also be reported to the National Institute for Minority Health and Health Disparities, which funds this research.

As part of the Data and Safety Monitoring Plan, a Data and Safety Monitoring Board (DSMB) will be established. The DSMB will consist of a minimum of two members including a Director of Research, to oversee the data collection aspects of the study, and a Clinical Director, to oversee the delivery of the intervention aspects of the study. Members will serve on the DSMB for a two-year term and will remain eligible for reappointment upon their term’s conclusion. The DSMB will review the research protocol, informed consent forms, and the Data and Safety Monitoring Plan; evaluate the progress of the trial, including timeliness of recruitment and data collection; consider relevant information external to the study; and make recommendations regarding initiation of participant recruitment, resolution of problems, and continuation, termination, or modification of the study.

### Statistical analysis

Initial ANOVA and Chi-square tests will be conducted to examine baseline equivalence of randomly assigned groups. Analyses with sex as a biological variable, including moderation and subgroup analyses, will be performed. Covariate entry (e.g., sociodemographics) will be decided based on model fit criteria, and sensitivity analyses will be performed for post-randomization risks.

In order to examine the added value of HS+PlayReadVIP in comparison to the standard HS model for the effectiveness phase of the study, we will focus on outcomes related to ERH and ECD where we expect the effects of standard HS to be substantially smaller than the integrated HS + VIP model. Impact analyses at each assessment timepoint will be conducted using ordinary least squares (OLS) regression in an intent-to-treat (ITT) framework, adjusted for baseline sociodemographic covariates. In addition, structural equation models (SEM) [31] will be used to examine family outcomes over time. This will allow us to examine whether initial levels and trajectories in parent well-being, parent-child interaction, and ECD vary between participants, and whether that variation is explained by assignment to HS + VIP or standard HS.

In order to examine implementation outcomes related to the HS+PlayReadVIP model, measure distribution, tests of normality, and confirmatory factor analysis will first be conducted, to create multi-indicator, multi-informant constructs where appropriate. To examine reach, effect sizes of differences between: 1) the study sample and clinic population; and 2) those who were eligible and enrolled vs. those who were eligible and declined will be determined by descriptive comparisons of multiple sociodemographic characteristics, including race/ethnicity, maternal age, child gender, and income. We will also use chi-square tests for independence to examine engagement (e.g., proportion of visits attended) in the HS+PlayReadVIP and standard HS groups. Perceptions of acceptability and appropriateness over time, between parent and provider participants, and between the HS+PlayReadVIP and standard HS groups will be examined using SEM. Finally, summary scores will be created for fidelity data across sessions (i.e., computing proportion of HS and/or VIP session components completed on average, and the interquartile range). Scores will be computed separately for PlayReadVIP and HS content for component analysis to understand whether differential implementation outcomes/challenges occur when HS+PlayReadVIP is implemented jointly.

### Mediation and moderation

In order to examine mechanisms and influences on relations between intervention group and ECD outcomes at 24 months, mediation and moderation analyses will be conducted. First, mediation analyses will be conducted according to principles set forth by MacKinnon [32] and Hayes, [33] using SEM with bias-corrected bootstrapping. Intervention impacts on parents, including psychosocial well-being, and ERH will be examined as potential mediators. Mediators will be examined separately, and only mediators on which HS+PlayReadVIP has a significant impact at 12M will be tested. Moderation analysis using OLS regression will be used to determine whether program impacts or implementation outcomes vary based on individual and intervention-related factors. Multiplicative interaction terms between group assignment and individual moderators, including family and child characteristics, as well as perceptions of acceptability and appropriateness.

### Missing data and attrition

Multiple imputation (MI) [34] will be used to replace item-level data using the rich array of concurrently collected variables and prior responses. If an entire measure/survey is missing, we will use chained equations with lagged (in time) variables [35] using all available information to build a large set of imputed datasets. Estimates will be combined using Rubin’s rules [34]. When data are Missing at Random, likelihood-based methods provide unbiased estimates of population parameters even when panels are incomplete. In addition, we will use attrition analyses [36] to examine whether attrition is related to baseline characteristics.

### Status and timeline

The protocol for this study is registered at clinicaltrials.gov with identification number NCT06941337. The first participant was recruited on June 9, 2025. To date, 194 participants have been enrolled in the study, with 35 in the implementation phase and 159 in the effectiveness phase. The 12-month follow-up assessments began on January 5, 2026. Recruitment for this study will be completed by January 2027, and the study’s primary completion date (i.e., completion of data collection) will be January 2029. Results from 12-month assessment are expected in mid-2028, with final results expected after completion in 2029.

### Ethical considerations

The safety and comfort of participants is a priority in this study. As such, all study procedures, informed consent forms, and recruitment materials have been approved by the NYUGSOM IRB (s24-01988). At the time of recruitment, participants are given detailed information about the study verbally and through a written informed consent form and briefer “key information” form in the participant’s native language (English of Spanish). During the consent process, trained study personnel read the informed consent form with the participant in detail, including the nature of the study, inclusion and exclusion criteria, a description of each assessment, and all risks and benefits. The contact numbers of senior study staff are listed on the consent form and will be pointed out to participants during this time. Participants are informed that participation is voluntary, that their decision to participate or not will not affect the care they receive, and that they can withdraw their enrollment at any time. Signed physical or electronic consent is obtained after all participant questions have been answered and it is clear they understand the study procedures.

The main ethical consideration in this randomized trial is participant and data confidentiality. Participant contact information is securely stored electronically on REDCap, a HIPAA-compliant secure web application that includes password protection and internal quality checks to identify data that appear inconsistent, incomplete, or inaccurate. Data will be periodically downloaded for backup purposes and stored on NYUGSOM managed shared drives with appropriate encryption and firewalls. Participants are assigned a unique study identifier (ID) which will be used for all assessments and data processing and analysis. Only approved study personnel have access to the database linking study IDs with participant contact information. All assessment data, including questionnaires, video-recordings, and other measure data, will be stored electronically on NYUGSOM managed shared drives with appropriate encryption and firewalls. Moreover, we have obtained a Certificate of Confidentiality (CoC) from NIH to protect the privacy of subjects in this research study. CoCs prohibit disclosure of identifiable, sensitive research information to anyone not connected to the research except when the participant consents or in a few other specific situations.

Additional ethical considerations include discomfort of adult or child participants due to embarrassment or discomfort in disclosing private information during interviews or in being observed during interactions with their child. In order to address this risk, participants are reminded that all portions of the assessments are voluntary and that they can skip any questions or portions with which they do not feel comfortable. Study personnel are trained to identify indicators of discomfort in both adults and children, and offer to answer questions, remind participants of the confidentiality of their information, and suggest skipping portions, taking a break, or discontinuing the assessment as appropriate

Lastly, because the HS and PlayReadVIP program providers at the NYU Langone Health Family Health Centers along with pediatric clinicians and clinic management will be participants for the implementation phase of the study, special considerations apply because they are employees. Employees may feel pressure to participate or to answer questions in a specific manner. Therefore, employee participants will be reminded that all information will be kept confidential. In addition, passive recruitment methods involving a non-supervisory recruiter will be used and the study team will ensure that all participants understand that participation is voluntary and will not impact the status of their employment in any way.

Any amendments to the protocol will undergo review by the NYUGSOM IRB and will be documented, including a justification for the change, by the study team. Significant protocol changes will also be noted in future publications of study outcomes.

## Discussion

Completion of study aims will advance preventive efforts to decrease poverty-related disparities in ECD through promotion of ERH by integrating of two low-cost, strengths-based ERH programs – HealthySteps and PlayReadVIP. This study will address gaps in our knowledge of how to integrate the programs, particularly for those with the greatest needs. It has the potential to result in transformative changes in policy and practice to prevent such disparities at the population-level, and findings will inform the scaling of ERH services in PPC by providing information about both the effectiveness of the integrated HS+PlayReadVIP model, as well as feasibility, acceptability, appropriateness, and participant adherence.

### Limitations of study design

Although the effectiveness-implementation hybrid RCT is a rigorous design, this study is not without limitations. First, because our study focuses on English and Spanish speaking families with increased social needs (i.e., HealthySteps Tier 3), findings may not generalize to other populations or to less intensive interventions meant for universal implementation. Second, because the study employs an active comparator or control arm, effect sizes demonstrating the added value of the combined intervention (HS+PlayReadVIP) may be small. Effect size differences may also be reduced due to spillover effects of HS+PlayReadVIP for families in the standard HS group through shifts in clinic culture and provider behaviors, as both interventions are being delivered in the same clinics. However, our implementation measures should capture such spillover effects, and planned moderation analyses will be particularly important for disentangling this question.

### Dissemination plans

Dissemination efforts will include presentation of findings for both academic research and clinical practice communities. Given the importance of both the potential implementation and effectiveness outcomes for future research examining ERH programs, findings from the current study will be of interest to researchers across multiple fields. Therefore, we plan to present findings at regional, national, and international conferences serving a variety of disciplines, including pediatrics and population health, as well as education, psychology, and other fields related to child development. We also plan to publish research findings in peer-reviewed journals using the Consolidated Standards of Reporting Trials (CONSORT) to ensure complete and accurate reporting. We will also follow NIH guidelines related to the public access of published manuscripts. Deidentified data will be shared after the primary completion date on the NICHD Data and Specimen Hub (DASH).

Additionally, as pediatric clinics begin to act on AAP and NASEM recommendations to implement and integrate ERH interventions, findings from this study can provide important guidance related to best practices for implementation, as well as understanding mechanisms of effectiveness to guide decision-making and advocacy efforts. As such, this trial is being included as a case study in *Integrating Early Relational Health Programs in Pediatric Primary Care: A Toolkit*, a freely-available best practices guide developed by the research team. The case study will be updated as results become available to provide a comprehensive example of the strategies outlined in the toolkit.

## Supporting information

S1 File SPIRIT checklist. (DOCX)

S2 File Approved protocol [37–58]. (DOCX)
