# Supplementary material for: Randomized single-blind study to examine the implementation and effectiveness of integrating evidence-based early relational health programs in pediatrics for families with low incomes: A clinical trial protocol
Source: PLoS One. 2026 Jul 24;21(7):e0354239. doi: 10.1371/journal.pone.0354239 (PMC13399281; doi:10.1371/journal.pone.0354239)
Supplement: S2 File — (DOCX) [file pone.0354239.s002.docx]

**S2. Appendix**

**HealthySteps + PlayReadVip effects on parenting and child development: A randomized implementation effectiveness trial**

| **Principal Investigator:** | Caitlin F. Canfield, PhD  Department of Pediatrics, NYU Grossman School of Medicine  462 First Ave, OBV A529, New York, NY 10016  Caitlin.canfield@nyulangone.org  212-562-2522 |
| --- | --- |
| **Additional Investigators:** | Elizabeth B. Miller, PhD  Department of Population Health, NYU Grossman School of Medicine  180 Madison Ave, 6^th^ Floor, New York, NY 10016  elizabeth.miller@nyulangone.org |
| **NYULH Study Number:** | *s23-00711* |
| **Funding Sponsor:** | [National Institute on Minority Health and Health Disparities (NIMHD)](https://era.med.nyu.edu/IRB/app/portal/smartform/read?project=com.webridge.entity.Entity%5BOID%5B09CAB398C82E5243BE0A213B205CD5F9%5D%5D&projectEditorView=com.webridge.entity.Entity%5BOID%5B2BFF71359DB164488A63C2D999C5F038%5D%5D)  National Institutes of Health  6707 Democracy Boulevard, Suite 800  Bethesda, MD 20892-5465 |
| **ClinicalTrials.gov Number** | Pending |

**Initial version:** 12/30/2024

**Amended:** 2/20/2025 – updated provider incentives, in line with request from clinic leadership

**Amended:** 3/26/2025 – fixed a typo related to family incentives

**Amended:** 5/30/2025 – updated recruitment protocol to include recruitment by phone

**Amended:**  9/4/2025 – added measures for the follow-up assessments

**Amended:** 2/9/2026 – added a newly available measure of bilingual language development

**Statement of Compliance**

This study will be conducted in accordance with the Code of Federal Regulations on the Protection of Human Subjects (45 CFR Part 46), any other applicable US government research regulations, and institutional research policies and procedures. The Principal Investigator will assure that no deviation from, or changes to the protocol will take place without prior agreement from the sponsor and documented approval from the Institutional Review Board (IRB), except where necessary to eliminate an immediate hazard(s) to the trial participants. All personnel involved in the conduct of this study have completed Human Subjects Protection Training.

**Table of Contents**

Protocol Summary 1

Schematic of Study Design 3

1 Key Roles 4

2 Introduction, Background Information and Scientific Rationale 4

2.1 Background Information and Relevant Literature 4

2.2 Rationale 6

2.3 Potential Risks & Benefits 8

2.3.1 Known Potential Risks 8

2.3.2 Risk of Use of Mobile Health Technology 9

2.3.3 Known Potential Benefits 9

3 Objectives and Purpose 10

3.1 Primary Objective 10

4 Study Design and Endpoints 10

4.1 Description of Study Design 10

4.2 Study Endpoints 11

4.2.1 Primary Study Endpoints 11

5 Study Enrollment and Withdrawal 11

5.1 Inclusion Criteria 12

5.2 Exclusion Criteria 12

5.3 Vulnerable Subjects 12

5.3.1 Risks and Benefits 12

5.4 Strategies for Recruitment and Retention 13

5.4.1 Use of DataCore/Epic Information for Recruitment Purposes 14

5.5 Duration of Study Participation 15

5.6 Total Number of Participants and Sites 15

5.7 Participant Withdrawal or Termination 15

5.7.1 Reasons for Withdrawal or Termination 15

5.7.2 Handling of Participant Withdrawals or Termination 15

5.7.3 Premature Termination or Suspension of Study 15

6 Behavioral/Social Intervention 16

6.1 Study Behavioral or Social Intervention(s) Description 16

6.1.1 Administration of Intervention 16

6.1.2 Procedures for Training Interventionalists and Monitoring Intervention Fidelity 17

7 Study Procedures and Schedule 17

7.1 Study Procedures/Evaluations 17

7.1.1 Study Specific Procedures 17

7.1.2 Standard of Care Study Procedures 18

7.2 Laboratory Procedures/Evaluations 18

7.3 Study Schedule 18

7.3.1 Screening 18

7.3.2 Enrollment/Baseline 19

7.3.3 Intermediate Visits 19

7.3.4 Final Study Visit 19

7.4 Concomitant Medications, Treatments, and Procedures 19

7.5 Justification for Sensitive Procedures 19

7.6 Prohibited Medications, Treatments, and Procedures 20

7.7 Prophylactic Medications, Treatments, and Procedures 20

7.8 Participant Access to Study Intervention at Study Closure 20

8 Assessment of Safety 20

8.1 Specification of Safety Parameters 20

8.1.1 Definition of Adverse Events (AE) 20

8.1.2 Definition of Serious Adverse Events (SAE) 20

8.1.3 Definition of Unanticipated Problems (UP) 21

8.2 Classification of an Adverse Event 21

8.2.1 Severity of Event 21

8.2.2 Relationship to Study Intervention 22

8.2.3 Expectedness 22

8.3 Time Period and Frequency for Event Assessment and Follow-Up 22

8.4 Reporting Procedures – Notifying the IRB 23

8.4.1 Adverse Event Reporting 23

8.4.2 Serious Adverse Event Reporting 23

8.4.3 Unanticipated Problem Reporting 23

8.4.4 Reporting of Pregnancy 24

8.5 Reporting Procedures – Notifying the Study Sponsor 24

8.6 Reporting Procedures – Participating Investigators 24

8.7 Study Halting Rules 24

8.8 Safety Oversight 24

9 Statistical Considerations 26

9.1 Statistical and Analytical Plans 26

9.2 Statistical Hypotheses 28

9.3 Analysis Datasets 28

9.4 Description of Statistical Methods 28

9.4.1 Safety Analyses 28

9.4.2 Adherence and Retention Analyses 29

9.4.3 Baseline Descriptive Statistics 29

9.4.4 Planned Interim Analysis 29

9.4.5 Additional Sub-Group Analyses 29

9.4.6 Multiple Comparison/Multiplicity 29

9.4.7 Tabulation of Individual Response Data 29

9.4.8 Exploratory Analyses 29

9.5 Sample Size 29

9.6 Measures to Minimize Bias 30

9.6.1 Enrollment/Randomization/Masking Procedures 30

9.6.2 Evaluation of Success of Blinding 30

9.6.3 Breaking the Study Blind/Participant Code 31

10 Source Documents and Access to Source Data/Documents 31

11 Quality Assurance and Quality Control 31

12 Ethics/Protection of Human Subjects 32

12.1 Ethical Standard 32

12.2 Institutional Review Board 32

12.3 Informed Consent Process 32

12.3.1 Consent/Assent and Other Informational Documents Provided to Participants 32

12.3.2 Consent Procedures and Documentation 32

12.4 Posting of Clinical Trial Consent Form 33

12.5 Participant and Data Confidentiality 33

12.5.1 Research Use of Data 34

12.6 Secondary Use of Stored Data 34

13 Data Handling and Record Keeping 35

13.1 Data Collection and Management Responsibilities 35

13.1.1 Data Collection Tools – Mobile Health Technology 35

13.2 Study Records Retention 36

13.3 Protocol Deviations 36

13.4 Publication and Data Sharing Policy 36

14 Study Finances 37

14.1 Funding Source 37

14.2 Costs to the Participant 37

14.3 Participant Reimbursements or Payments 37

15 Study Administration 37

15.1 Study Leadership 37

16 Conflict of Interest Policy 37

17 References 39

18 Attachments 50

**List of Abbreviations**

| AE | Adverse Event/Adverse Experience |
| --- | --- |
| CFR | Code of Federal Regulations |
| CRF | Case Report Form |
| CSOC | Clinical Study Oversight Committee |
| DCC | Data Coordinating Center |
| DHHS | Department of Health and Human Services |
| DSMB | Data Safety Monitoring Board |
| FFR | Federal Financial Report |
| FWA | Federalwide Assurance |
| GCP | Good Clinical Practice |
| HIPAA | Health Insurance Portability and Accountability Act |
| ICF | Informed Consent Form |
| IRB | Institutional Review Board |
| ISM | Independent Safety Monitor |
| MOP | Manual of Procedures |
| N | Number (typically refers to participants) |
| NIH | National Institutes of Health |
| OHRP | Office for Human Research Protections |
| OHSR | Office of Human Subjects Research |
| PI | Principal Investigator |
| QA | Quality Assurance |
| QC | Quality Control |
| SAE | Serious Adverse Event/Serious Adverse Experience |
| SOP | Standard Operating Procedure |
| US | United States |

| AE | Adverse Event/Adverse Experience |
| --- | --- |
| CFR | Code of Federal Regulations |
| CRF | Case Report Form |
| CSOC | Clinical Study Oversight Committee |
| DCC | Data Coordinating Center |
| DHHS | Department of Health and Human Services |
| FFR | Federal Financial Report |
| FWA | Federalwide Assurance |
| HIPAA | Health Insurance Portability and Accountability Act |
| ICF | Informed Consent Form |
| IRB | Institutional Review Board |
| MOP | Manual of Procedures |
| N | Number (typically refers to participants) |
| NIH | National Institutes of Health |
| OHRP | Office for Human Research Protections |
| OHSR | Office of Human Subjects Research |
| PI | Principal Investigator |
| QA | Quality Assurance |
| QC | Quality Control |
| SAE | Serious Adverse Event/Serious Adverse Experience |
| SOP | Standard Operating Procedure |
| US | United States |
| ERH | Early Relational Health |
| ACE | Adverse Childhood Experience |
| PCE | Positive Childhood Experience |
| EBPI | Evidence-Based Preventive Intervention |
| APA | Academic Pediatric Association |
| VIP | Video Interaction Project |
| HS | HealthySteps |
| CORNET | Continuity Research Network |
| HS NO | HealthySteps National Office |
| ERIC | Expert Recommendations for Implementing Change |
| OSF | Open Science Framework |

# Protocol Summary

| Title | HEALTHYSTEPS + PLAYREADVIP EFFECTS ON PARENTING AND CHILD DEVELOPMENT: A RANDOMIZED IMPLEMENTATION EFFECTIVENESS TRIAL |
| --- | --- |
| Short Title | HealthySteps + PlayReadVIP |
| Brief Summary | This study aims to investigate the effectiveness and implementation outcomes for the integration of two evidence-based practices and interventions (EBPIs) within pediatric primary care settings: HealthySteps (HS), which provides parenting support, and PlayReadVIP, which promotes relational health through video coaching. Families will be randomized to receive either the integrated HS + PlayReadVIP model or traditional HS, and we will examine impacts on parenting, parent-child relationships, and child development, as well as feasibility, acceptability, and appropriateness of the integrated intervention. |
| Phase | Phase 3 |
| Objectives | This study aims to:  1) Assess the effectiveness and implementation of combining the HealthySteps (HS) parenting support model with PlayReadVIP to improve parenting and relational health outcomes.  2) Evaluate mechanisms of intervention impact, including mediation through social determinants of health and moderation by implementation outcomes. |
| Methodology | Randomized hybrid effectiveness-implementation trial |
| Endpoints | Parenting practices, parent-child relationships and child development, as well as feasibility, acceptability, appropriateness, and reach. |
| Study Duration | 5 years |
| Participant Duration | 2 years |
| Duration of behavioral intervention | 2 years |
| Population | 500 parent/infant dyads + 25 care providers |
| Study Sites | NYU Langone Health - Sunset Park Family Health Centers |
| Number of participants | 1025 participants |
| Description of Study Intervention/Procedure | The intervention being evaluated in this study integrates two evidence-based programs, HealthySteps (HS) and PlayReadVIP, into a combined model HS+PlayReadVIP to promote early childhood development and parent-child relational health. The integrated intervention will be implemented during routine pediatric well-child visits for families identified as Tier 3 (increased risk and reduced resources) by the HealthySteps Specialist. |
| Reference Therapy | Standard HealthySteps program |
| Key Procedures | N/A – No procedures. At enrollment, 12m, and 24m:  Parent Surveys: Parenting practices, mental health (depression, anxiety).  Child Assessments: Developmental milestones, school readiness, behavioral outcomes.  Observations: Parent-child interactions during study visits.  Medical Records: Updates on health and developmental progress.  All assessments will be conducted by trained research assistants blinded to intervention status. |
| Statistical Analysis | Effectiveness Outcomes: Comparison of parenting practices and relational health between groups using multivariate regression, structural equation models (mediation) and other statistical analyses appropriate for longitudinal data.  Implementation Outcomes: Descriptive statistics and thematic analysis of qualitative data |

# Schematic of Study Design

Approximately 1000 **individuals** -500 parent-infant dyad

dyad)

Randomize

Parent surveys, demographic information, and baseline measures of parenting assets, vulnerabilities

Families receive intervention (HS+PlayReadVIP or standard HS)

Parenting, ERH, and ECD outcomes, as well as intervention acceptability assessed through surveys, observations, and direct assessments

Parenting, ERH, and ECD outcomes, as well as intervention acceptability assessed through surveys, observations, and direct assessments

Qualitative interviews focusing on family needs, resources, and context

Baseline

0–24 months

Child age 12m; 6+ months post-enrollment

Child age 24m; 18 months post-enrollment

End of study (child age 24m)

Families with infants ≤6M, randomized into HS+PlayReadVIP or standard HS groups

Approximately **25** individual care providers

Acceptability & appropriateness of intervention; clinic climate & processes

Qualitative interviews of implementation outcomes

Acceptability & appropriateness of intervention; clinic climate & processes

# Introduction, Background Information and Scientific Rationale

## Background Information and Relevant Literature

Poverty and racism are linked to disparities in early child development (ECD), a core contributor to long-term physical and mental health.^1,2^ These disparities begin early in life,^3,4^ widen as children age,^1^ persist across the lifespan,^1,2^ and stem from broad contextual factors resulting in inequality of resources and opportunity.

Such inequality increases the likelihood of adverse childhood experiences (ACEs) and decreases the likelihood of positive childhood experiences (PCEs). Two interrelated frameworks illustrate how ACEs and PCEs impact child development through ***a key common pathway*** of early relational health (ERH; parent-child relationship quality, structuring of the home environment, responsivity/cognitive stimulation^5^). The family stress model^6^ posits that increased parent vulnerabilities (e.g., maternal depression) negatively affect relationship quality elements of ERH (e.g., harsh interactions), while the family investment model^7^ posits that reduced parent assets (e.g., learning materials) negatively affect cognitive stimulation elements of ERH (e.g., reading aloud).

Enhancing ERH by ***both addressing ACEs and promoting PCEs*** is critical for mitigating disparities in ECD. Evidence-based preventive interventions (EBPIs) designed to reduce these disparities have increasingly been located in pediatric primary care due to its population-level reach, frequent visits, trusted relationships, and potential for low cost. Guided by American Academy of Pediatrics (AAP) recommendations and healthcare redesign efforts,^8,9^ such EBPIs utilize routine well-child visits to address ACEs or to promote PCEs through parenting programs that provide anticipatory guidance and provision of learning materials. Although these programs have shown some efficacy in reducing disparities in ECD, their impact may be limited because they do not have the capacity to concurrently and directly target both ACEs and PCEs.^10-12^ Therefore, integrating programs that can cohesively do both within pediatric primary care provides a novel, scalable method for reducing disparities in ECD. However, knowledge gaps related to dissemination and implementation (D&I) of integrated EBPIs represent a ***key barrier to improving implementation effectiveness and impact***.

We propose a novel integration of two exemplar, AAP-recommended EBPIs delivered at pediatric well visits:

**1) HealthySteps (HS)** utilizes a stepped-care approach including universal screening for ACEs and child developmental delays. Families with increased concerns also receive customized support and referral for additional services. HS is rapidly becoming standard of care in pediatrics with implementation in 25 states and over 200 sites. Whereas HS has impacts on family vulnerabilities (e.g., maternal depressive symptoms^13,14^), it has limited impacts on PCEs, which are not addressed through direct HS services. Thus, even as screening and services to address ACEs have become standard of care in pediatrics, ***this suggests the need for additional strategies to target ERH and child outcomes in order to improve effectiveness*.**

**2) PlayReadVIP** has the potential to address this gap through a focus on PCEs by video-recording the parent and child playing with a toy or reading a book with immediate, real-time review and feedback to identify and reinforce strengths in the interaction. PlayReadVIP has beneficial impacts on parenting assets (e.g., learning materials^15^), ERH/cognitive stimulation (e.g., reading aloud^16^), and child outcomes.^17^ PlayReadVIP’s low cost and strong evidence base have resulted in increased implementation in pediatrics, with 13 sites in 3 cities.

The overarching objective of this protocol, then, is to understand both the effectiveness and practical implementation of the HS+PlayReadVIP model in real-world pediatric primary care environments. Currently, implementing early childhood interventions like HS+PlayReadVIP in pediatric primary care presents several challenges; such as time limitations, limited funding, staffing Issues, lack of training , not reaching the diverse patient polulation and not addressing social determinants directly. This protocol focuses on 2 principle aims that will asses the impact of the integrated HS+PlayReadVIP model on various outcomes : Aim 1: Assess the effectiveness of the HS+VIP model on parent-child relational health, parent mental health, and parenting behaviors compared to standard HealthySteps (HS). And Aim 2: Evaluate the implementation outcomes of the HS+VIP model, including feasibility, acceptability, appropriateness, reach, engagement, and fidelity. Working on these challenges is crucial for the successful implementation and sustainability of early childhood interventions in pediatric primary care settings.

The overall goals are :

1. Aim 1: Determine whether the integrated HealthySteps (HS) and Video Interaction Project (VIP) model has greater impacts on parenting and early child development (ECD) compared to standard HS alone.
2. Aim 2: Understand how and why the integrated HS+VIP model works by examining the mechanisms driving its impacts and the contexts influencing its implementation.

Together, Aim 1 and Aim 2 will:

- Demonstrate the value of integrating evidence-based preventive interventions in pediatric primary care to address disparities in child development.
- Generate actionable knowledge about how to implement and sustain such integrated models effectively in diverse healthcare settings.

By addressing these aims, the study seeks to provide comprehensive insights into both the effectiveness and practical implementation of the HS+VIP model in real-world pediatric primary care environments.

This project will be led by Dr. Caitlin Canfield, a developmental psychologist with a primary appointment as compensated faculty in the Department of Pediatrics at NYU Grossman School of Medicine.

Primary endpoints: Parenting practices, parent-child relationships and child development.

## Rationale

The rationale for the study is grounded in the need to address disparities in Early Child Development (ECD) by integrating effective and scalable interventions within pediatric primary care.

**Rationale for Implementation and Study of the Integrated HS+PlayReadVIP Model.Aim 2**

**I. Early Child Development (ECD): A Key Health Domain Across the Lifespan.** ECD, including cognition, language skills, and social competence, is widely recognized as a core domain of child health,^20^ making major contributions to children’s educational achievement^21^ and economic attainment across the lifespan,^1,2^ as well as to adult physical and mental health, with links to reductions in risk factors for cardiovascular and metabolic disease, stress, and aggression.^22,23^ As a result, the United Nations has targeted ECD as 1 of 17 Sustainable Development Goals for 2030.^24^

**II. Poverty and Racism: Central to Disparities in ECD.** Poverty and racism in the U.S. are strongly linked to disparities in ECD through impacts at the societal, system/ provider, and community/family levels (Fig. 1; adapted from NIMHD Health Disparities Framework).^25-30^ Nearly 40% of young children ages 0-5 years live in poverty (<50%-100% of Federal Poverty Line [FPL]) or with low income (100-200% of FPL).^31^ Further, growing up in households with low incomes is much more common for Latinx (53%) and Black/African-American (58%) children compared to White children (26%).^31^ ECD disparities related to poverty and racism begin early in life and widen as children age.^1,2^ As early as 9M, infants in families with low incomes show cognitive differences,^30^ and by 18M, differences in vocabulary and language processing emerge.^3^ ~40-50% of children in families with low incomes have reduced school readiness in at least one domain,^32^ leading to gaps in reading (Black-White gap: .31 *SD*; Latinx-White gap: .56 *SD*; income gap: 1.06 *SD*) and math levels (Black-White gap: .55 *SD*; Latinx-White gap: .67 *SD*; income gap: 1.17 *SD*),^32-34^ in conjunction with increased behavior problems (e.g., aggression; income gap: .74 *SD*) and inattention that are critical barriers to learning.^35^

**III. Mechanisms of Disparities.**

*A. Adverse Childhood Experiences (ACEs) and Positive Childhood Experiences (PCEs).* *ACEs* are traumatic events that occur in childhood (e.g., family violence, child abuse/neglect, parental psychopathology, substance abuse).^36^ ACEs are common (61% of adults have experienced 1+ ACEs; nearly 17% have had 4+ ACEs^37^), and disproportionately impact racial/ethnic minority families (3+ ACEs: 17% vs. 10%^38^) and those with low incomes (3+ ACEs: 13% vs. 5%^38^). Due to prolonged activation of the stress-response system (i.e., toxic stress^39^), ACEs have costly long-term consequences for brain development^40-43^ and lasting negative effects on health and well-being,^44-49^ including attention, decision-making, and emotion regulation.^38,49^ *PCEs* are experiences essential for child development, social emotional competencies, and mental health, and include supportive and responsive relationships; living, playing, and learning in safe, stable environments; and opportunities for social engagement and connectedness.^50^ Children from racial/ethnic minority families (6+ PCEs: 38% vs. 54% of White children) and homes with low incomes (6+ PCEs: 40% vs. 66%) experience fewer PCEs.^51^ Importantly, *PCEs are not simply the absence of ACEs***.** Rather, they represent resources and socioecological factors that allow children to thrive and that may be particularly valuable in the context of ACEs, providing a critical buffer that makes it possible for children to flourish in the context of increased risk.^9,51-52^ For instance, both family resilience (parent-child connection, coping) and supportive parenting (reading, singing, sharing meals) have been linked to positive child outcomes, including fewer social-emotional problems, lower risk for developmental delay, and increased persistence and self-regulation, even in the context of 4+ ACEs.^53,54^ At the same time, for families without ACEs, lack of PCEs is associated with levels of child risk comparable to families with ACEs.^53,54^ Thus, screening and services to address ACEs (often standard of care in pediatrics) are likely insufficient to protect children from the impacts of adversity, still less to thrive academically and socially, unless there is also simultaneous promotion of PCEs.

*B. Early Relational Health (ERH).*

*B1. A Key Common Pathway****:*** ERH (Fig. 2), defined as parent-child relationship quality, structuring of the home environment, and responsive/cognitively stimulating practices,^5^ represents a key common pathway by which ACEs and PCEs impact child development.^55^ Importantly, ERH actively promotes skills children need to respond to adversity in a healthy, adaptive manner, including self-regulation, adaptability, and mastery orientation.^56,57^ ACEs negatively affect ERH and ECD through ecological factors and parenting capacities that are barriers to ERH, while PCEs can be considered as both ERH itself and underlying parenting assets that buffer impacts of ACEs and proactively promote ECD and later social and cognitive skills.^9^

*B2. Underlying Frameworks****:*** ERH can be conceptualized as a convergent outcome within two interrelated frameworks with broad theoretical and empirical support (Family Stress and Family Investment models; Fig. 2), supporting its salience as a target for early preventive intervention.^5^

*Family stress model* (FSM):^6^ This posits that inequality of resources and opportunity impact child development^58^ through parent emotional/relational distress^59^ and consequent effects on relationship quality components of ERH (e.g., increased harsh interactions). FSM impacts on ECD (especially child social-emotional competence) are subsequently associated with long-term trajectories of health and well-being (e.g., employment, relationship stability).^36^ The FSM suggests that decreasing ACEs by addressing parent and other psychosocial stressors,^55,60-62^ particularly for families with few resources, can promote ERH and better child outcomes. *Family investment model* (FIM):^7^ This posits that inequality of resources and opportunity impact child development by limiting resources and parent assets, particularly time and money, thereby limiting capacity for provision of learning materials (books, toys), and reducing prospects for 1-on-1 interactions that promote children’s learning skills, with consequent reduction in the responsivity/cognitive stimulation components of ERH (e.g. reading, teaching, and play^16,62-64^). Similar to FSM, FIM impacts on ECD (cognitive, language and social-emotional competence^9,52,65-66^) are associated with long-term trajectories of health and well-being. The FIM suggests that increasing PCEs by supporting ERH and parenting assets can buffer ACEs and enhance outcomes.^68^

**IV. Evidence-Based Preventive Interventions (EBPIs) Targeting ERH and ECD.**

*A. EBPIs May Address ACEs or Promote PCEs***.** EBPIs are research-based, theory-driven programs that aim to preclude disparities by addressing underlying mechanisms before problems emerge.^69^ There is strong evidence that EBPIs targeting either the FSM (i.e., ACEs) or FIM (i.e., PCEs) can have important impacts on ERH and ECD.^68,70-2^ Such EBPIs often implicitly address both ACEs and PCEs, either by targeting ACEs that are barriers to PCEs (i.e., indirectly targeting PCEs), or by enhancing PCEs that have been shown to have reciprocal impacts on specific ACEs (i.e., indirectly targeting ACEs). For instance, the Family Check-Up home visiting program reduces maternal depression (ACE), which in turn increases supportive parenting (PCE), resulting in reduced child behavior problems.^72^ However, most such programs do not *simultaneously and directly* address ACEs and promote PCEs, and therefore may not be adequate to fully reduce disparities or comprehensively impact ECD, particularly for families with the fewest resources.^5,73^ *Theoretical and empirical considerations together, therefore, suggest that integrated EBPIs that target both ACEs and PCEs have the potential to address this gap*.

Thus, we hypothesize that the integrated HealthySteps (HS) and PlayReadVIP model (HS+PlayReadVIP) will promote early relational health and child development outcomes by simultaneously addresses ACEs and promotes PCEs, offering a comprehensive approach to supporting parenting and enhancing ECD outcomes.

**Rationale for implementation in pediatric primary care.**

**I. Pediatric Primary Care as a Platform for EBPIs.** Pediatric primary care is an ideal platform for EBPIs with potential for nearly universal reach and population-level scaling,^12,74-75^ and frequent contact starting at birth that is maintained via preventive care (immunization, screening; 13+ visits by age 5). Further, there is opportunity to utilize existing infrastructure, implement at relatively low cost, and build on trusting relationships with providers to foster engagement.^76^ Recent American Academy of Pediatrics (AAP) policy statements (e.g., toxic stress)^8,9^ therefore advocate for implementation of EBPIs in primary care, and policy leaders (Center for the Study of Social Policy, InCK Marks) have recommended pediatric primary care redesign efforts supporting this.^77,78^ Yet, addressing ACEs and promoting PCEs requires offering multiple, independent EBPIs, leading to barriers including time, cost, and engagement of parents across programs.^79-82^ Therefore, development of integrated EBPI models that target ACEs and PCEs together may provide a novel, scalable method for reducing ECD disparities. *Importantly, however, knowledge gaps related to dissemination and implementation (D&I) of integrated EBPIs represent a key barrier to improving impact and implementation effectiveness*.

**II. Collaborative Models Support Implementation of EBPIs in Pediatric Primary Care.** Two broadly categorized models developed and studied over the last several decades have been identified as useful strategies to support EBPI integration and implementation and are considered in this application.^83-85^ 1) In *coordinated approaches*, primary care physicians and specialists (e.g., behavioral health) at different sites collaborate extensively to provide care and refer families to community or other professional resources as needed. 2) In *co-located approaches*, primary care physicians and specialists practice at the same site and work as a team to address patient needs, including in-house and/or external referrals. These two models have been applied to collaborative behavioral health services in pediatric primary care (e.g., ADHD) and have shown implementation feasibility and effectiveness.^86,87^ However, the effectiveness of these strategies for integrating 2+ EBPIs in pediatric settings has not been systematically studied. This application builds on integrated health service research to identify optimal strategies to integrate coordinated and co-located EBPIs in pediatric care.

**III.** **Challenges and Strategies to be Considered in Implementation Model Design.** Despite the inherent utility for service delivery and potential for scaling and reach, implementing integrated EBPIs in pediatric primary care comes with challenges that need to be considered during the design phase. Prior implementation and service integration research of EBPIs in diverse service contexts^96-103^ identified **two key challenges** related to implementing EBPIs in pediatric primary care: 1) *limited human resources/capacity*: staff time and capacity for engaging parents from underserved communities with competing priorities and diverse cultural beliefs; scarcity of behavioral health specialists; limited pediatrician-patient interaction time in well-child visits; and 2) *clinical workflow modification and efficiency*: staff buy-in; clarity on their role and care model for the added EBPIs; service information communication within the care team. **Several strategies** have been proposed to address these challenges, including *task-shifting* (training non-medical professionals/care team support staff to take on the EBPI implementation roles to promote ERH and address ACEs), *task-sharing collaborative strategies* (sharing multiple EBPI tasks with available care team structure and resources), and *system-level intervention strategies* (strengthening providers’ technology system [in e-materials, e-communication] to promote service delivery efficiency and quality) to strengthen providers’ capacity and build clinic readiness/human resources. And *care team engagement and education strategies* (engaging care team in clinical workflow, quality monitoring, and implementation guideline design; staff training and coaching; regular meetings to promote staff buy-in and information sharing) and *partnership strategies* (care team member engagement in developing communication and collaboration strategies) to develop a clinical workflow that supports EBPI delivery. However, while these challenges and strategies may apply broadly, there has been limited study of D&I of integrated EBPI models, and it is unclear whether such integration may in itself address challenges related to EBPI implementation, or whether new barriers and facilitators specific to integrated models may emerge. This application will fill this gap by building on prior work, including our own prior study defining recommended delivery and implementation processes, as well as strategies for implementing integrated EBPIs during routine pediatric care through collaboration with pediatric providers across diverse sites.

Thus, we hypothesize that pediatric primary care will provide a feasible and acceptable platform for implementation of the HS+PlayReadVIP model.

## Potential Risks & Benefits

### Known Potential Risks

As this is a study of an intervention, the integrated HS+PlayReadVIP model, aimed to promote parent-chid relational health, parent mental health, and parenting behavior from birth to 24 months, compared with standard HS delivery. minimal risk to participants is expected.

Potential risks include:

1. Possible violation/loss of confidentiality
2. Anxiety or distress during assessment procedures due to discussion of sensitive personal information during surveys and in relation to their child’s developmental assessments
3. Concern about identification of child abuse or neglect, knowledge of an assault/exposure to domestic violence, criminality, or sensitive personal information.

Strategies to minimize risks:

1. *Violation/loss of confidentiality*: All physical paper records obtained from subjects, including questionnaires, video-recordings, and measures data, will be kept in locked file cabinets in locked rooms. In addition, all data will be de-identified prior to entering into data servers by removing subjects’ names and personal and demographic information and assigning them identification numbers. Electronic data will be stored in REDCap. Password protection, appropriate firewalls, and encryption systems will be in place to ensure privacy of information and the security and integrity of the electronically stored data. Cross-reference files (linking personally identifiable data with identification numbers) will only be made available to the PI and a limited number of research personnel if needed (e.g., for those staff responsible for merging information on sample members across data sources). For other team members conducting analyses, data will only be provided using the confidential ID numbers. All data will be reported in aggregate form, without identifying information or individual cases. Project staff will receive training on confidentiality, including data collection, data management, and reporting procedures, as well as on HIPAA-required procedures to ensure patient data confidentiality.
2. *Anxiety or distress due to discussion of sensitive topics*: Participants will be provided with contact information of the MPIs and their designees in case there is anything about the assessment or intervention procedures or their participation that is unclear, or that they do not understand, or they have questions or wish to report a research-related problem. Should the individual feel uncomfortable discussing his/her concerns with the project personnel, or about their rights as a research participant, contact information of the NYUGSOM IRB will be listed as advocates for their concerns.

Although unexpected, parents may feel some discomfort or anxiety due to questions that we ask or during child developmental assessments. However, all portions of the study are voluntary and families may elect to not answer questions or participate in measurements as they choose. In addition, families may withdraw from the study at any time without jeopardizing the care that they receive, and will be given answers to any questions that could arise throughout their participation in an effort to ease any discomfort or anxiety. Should parents experience an adverse reaction to the assessments and require medical and/or psychological assistance, project staff members will be trained to evaluate the situation and, if necessary, make appropriate referrals. Referrals will be made within the SPFHC medical home, and as needed to community based low-cost, high-quality service agencies on pre-existing processes for referral at the site.

1. *Concern about identification and reporting of child abuse/neglect, domestic violence, or other sensitive personal information*: We are required to disclose to the responsible authorities information we receive from subjects about child or other-family member maltreatment; and especially, intended physical harm and neglect to potential victims. Child maltreatment is defined as physical or mental injury, sexual abuse or exploitation, negligent treatment, or maltreatment of a child by a guardian, under circumstances that indicate that the child’s health or welfare is harmed or threatened. It is our legal and ethical responsibility to inform authorities and to protect the well-being of potential victims. Also, evidence of physical injury or neglect to a child (e.g., child abuse) or to an adult (e.g., domestic violence, elder abuse) caused by other-than-accidental means will be reported to the appropriate authorities. This procedure is explained to potential subjects both verbally and on the consent forms.

Additional Specific Considerations

1) Risks to pediatric participants: Minimal risk to child participants is expected. Assessment visits can result in boredom, performance anxiety, or fatigue. However, most children enjoy study assessment procedures which are designed to be experienced as play; participants may elect to withdraw at any time without penalty, and careful training will be provided to interviewers to recognize when children are refusing to participate in the assessments. Similarly, parents may become bored or distressed as a result of completing questionnaires and interviews about their child’s development. Examiners will be trained to provide appropriate support and follow-up, and personnel supervising assessments will be able to provide referrals should significant concerns about a child’s development arise. In addition, examiners will be trained to provide necessary logistical support to parents during assessments (e.g., if parents cannot read the questionnaires due to literacy levels). As with all studies, there are risks associated with breaches of confidentiality; however, our team will implement all Institutional Review Board (IRB) procedures approved for this study to minimize this risk.

2) Risk to care providers: HS+PlayReadVIP and other care providers may feel pressured to participate or to answer questions in a specific way. To mitigate this risk, passive recruitment methods involving a non-supervisory recruiter will be used and participants will be informed that all information provided will be kept confidential. In addition, interviewers will receive specific training to recognize when interviewees are feeling uncomfortable answering questions, and providers will be reminded that they can skip any questions they prefer not to answer. Finally, the study team will ensure that all participants understand that participation is voluntary and will not impact the status of their employment.

3) Alternative treatments and procedures: This is a preventive intervention. There are no alternative treatments and procedures. All families will receive standard pediatric care, including all screening and referral for all services as indicated in routine clinical practice. Families may receive the standard HealthySteps program, regardless of participation in the study, as they will have been screened as eligible.

### Risk of Use of Mobile Health Technology

NYULH MCIT-managed iPhones and iPads. These devices will be used in accord with the Terms of Service (TOS) and/or the End User License Agreements (EULA) provided by the product or device vendor. Use of such products and devices may result in loss of privacy and risk of breach of confidentiality. These products and devices will only be used to collect study data with IRB approval and if the subject has agreed to all applicable Terms of Service and EULAs. The participant will be advised to read the full EULA or TOS before agreeing to use the product. Any risks associated are outlined in the informed consent.

### Known Potential Benefits

The overall risk involved in the project is considered to be minimal, whereas the information gained could greatly improve our knowledge base about how to effectively integrate interventions in pediatric primary care to address heterogeneity in patient needs. Parents will complete standardized interviews and questionnaires about their own well-being, and their children’s development and behavior and their experience in the target intervention. Past research indicates that most parents appreciate having their attention drawn to the child's behavior and also being given an opportunity to talk about how they parent. Parents appear to enjoy the experience, which gives them a chance to discuss their own and their children's issues. Moreover, any positive (atypical) surveillance, screening, or evaluation results with clinical relevance will be discussed with the child’s parents and health provider, and the project will offer (and help facilitate) a referral for an Early Intervention evaluation up to age 3 years as needed. In addition, measures of maternal depression and parenting stress may lead to identification of parents in need of further evaluation and possible treatment; these parents will be offered referral for mental health services.

# Objectives and Purpose

The primary objective of this study is to evaluate the effectiveness and implementation of an integrated HealthySteps (HS) and Video Interaction Project (VIP) model (HS+VIP) in pediatric primary care to address disparities in Early Child Development (ECD).

## Primary Objective

There are two primary objectives of this study:

**Aim 1. Implement the optimized integrated HS+VIP model (formalized in Aim 1), and conduct an effectiveness-implementation study (Hybrid I design).**

1. Examine effectiveness of the integrated HS+VIP model, including parent mental health, parenting behavior, and early child development outcomes.
2. Examine implementation outcomes, including reach, appropriateness, acceptability, feasibility, and fidelity, using an integrated RE-AIM^18^ and CFIR^19^ framework.

**Aim 2. Examine health disparities mechanisms underlying effectiveness-implementation outcomes.** Using a mixed methods design, we will examine whether HS+VIP impacts are 1) mediated by impacts on parent assets/vulnerabilities and ERH or 2) moderated by CFIR contextual factors or RE-AIM domains.

# Study Design and Endpoints

## Description of Study Design

**Overall Study Design:**

Randomized Controlled Trial (RCT): 500 families will be enrolled between birth and 6 months of child age and randomized to 1) HealthySteps+PlayReadVIP or 2) standard HealthySteps; with intervention delivery and assessments of parenting assets/vulnerabilities, parenting practices/relationships and child development from enrollment to child age 2 years.

**1. Setting:** Enrollment, randomization, and intervention delivery will take place at the NYU Langone Sunset Park Family Health Centers (SPFHC) pediatric clinic. The SPFHC is a federally-qualified health center, consisting of co-located prenatal and pediatric clinics with 10 pediatric providers (nine pediatricians; one nurse practitioner) and designated space for intervention delivery and assessments. It is across the street from NYU Langone Hospital–Brooklyn (NYULHB). NYULHB has ~4,000 births/year with 25-30% pediatric follow-up at the SPFHC. In total from July 2021-June 2022, the SPFHC served 3,503 children ages 0-3 (84% Hispanic, 10% Black/African-American, 3% Asian). In a recent needs assessment, 54% of families surveyed (*n*=401) reported multiple needs related to ACEs and parenting.^137^ 96% of these families had insurance coverage through Medicaid or CHIP.

**2. Sampling:** A consecutive sample of 620 parent-infant dyads presenting to HCC pediatric clinic will be assessed for eligibility, with ~500 families enrolled (expected ~5% not meeting eligibility and refusal rate ~10-15%). Feasibility is based on the current rate of target-age dyads (~1000 newborns/year presenting for care at SPFHC), and will build upon processes developed for ongoing intervention studies at SPFHC and elsewhere. In addition, program and other care providers will be approached through passive recruitment methods to complete surveys and interviews related to program implementation.

**3. Eligibility criteria:** Parent/infant dyads enrolled between birth and 6 months. Specific inclusion/exclusion criteria are given in Section 4.

**4. Intervention:** HealthySteps+PlayReadVIP Additional details given in Section 6.

**5. Assessments and data collection:** For all participants, we will administer assessments at baseline, 12 months, and 2 years to measure outcomes (e.g., parenting assets/vulnerabilities, parenting practices/parent-child relationship, child development) and covariates (e.g., socio-demographics, biological risk). In addition, care providers will be asked to complete surveys and interviews at ther 12 month and 2 year assessment time points. All assessments will be completed by highly trained research assistants blind to group/intervention status. Additional details given in Section 7.

## Study Endpoints

### Primary Study Endpoints

Parent mental health, parenting practices, parent-child interaction, parent-child relationship, social determinants of health, child development, intervention feasibility, acceptability, reach, and participant engagement. These outcomes will be measured through surveys, interviews, parent-child observations, and direct assessments of child development (see Section 6 for additional details on specific measures).

### Secondary Study Endpoints

# Study Enrollment and Withdrawal

Study Enrollment and Withdrawal will include the following:

Enrollment:

- Participants will be parent-infant dyads and care providers recruited from the NYU Langone Sunset Park Family Health Center pediatric clinics.
- A total of 500 dyads and 25 care providers will be enrolled for the randomized controlled trial.
- Families will undergo baseline assessments, with follow-ups at multiple time points to evaluate parenting practices and relational health outcomes.

Withdrawal:

- Participants may withdraw at any time without penalty.
- Withdrawn participant’s data will be excluded from further analysis unless they have completed at least one follow-up.

In addition,

- Clinics or stakeholders may opt out at any stage.
- Data collected before withdrawal will be retained for aggregated implementation analysis.

## Inclusion Criteria for parent-child dyads

In order to be eligible to participate in this study as a parent/child dyad, including one parent and one child, the dyad must meet all of the following criteria:

1. Parent is a biological parent or legal guardian and at least 18 years of age
2. Infants must be ≤6 months old at the time of enrollment.
3. Parents/guardians must speak either English or Spanish to ensure they can fully engage with the intervention and assessments.
4. Families must be identified as Tier 3 HealthySteps participants, indicating higher levels of need based on clinic screening for factors such as:Adverse Childhood Experiences (ACEs) and parenting challenges or vulnerabilities.
5. Parent is able to willing to provide consent for their own and their child’s participation.

## Exclusion Criteria for parent-child dyads

An individual who meets any of the following criteria will be excluded from participation as a parent/child dyad in this study:

1. Non-singleton child
2. Parent is unable to provide consent
3. Parent does not speak English or Spanish, as study materials (e.g., questionnaires) are available in those languages
4. Parent has a severe medical or psychiatric impairment (e.g., intellectual disability, psychosis) that would interfere with study participation
5. Parent has plans to discontinue care at NYU Langone Health - Sunset Park Family Health Centers

## Inclusion and Exclusion Criteria for care providers

All care providers will be eligible to participate, with no other inclusion/exclusion criteria applied.

## Vulnerable Subjects

**Children**

This research study will enroll children. Parent participants will consent for their own and their children’s participation in the study. Because children will be too young to formally assent for their own participation, careful training will be provided to staff to recognize when children are refusing to participate for the direct assessments. The Project PI is a developmental psychologist and faculty in the Division of Developmental-Behavioral Pediatrics. The PI and Co-PI have extensive experience conducting intervention research with very young children. The combination of these experiences allows them substantial flexibility to examine the mechanisms by which early childhood environments and policies affect children and their families in areas as diverse as pediatric primary care, early childhood education, parenting, and program evaluation.

**NYULH Employees**

This study will include employees of the NYULH Family Health Centers, including pediatric primary care providers and PlayReadVIP and HealthySteps providers. Involvement of employees is necessary in order to understand the feasibility and acceptability of the integrated HealthySteps+PlayReadVIP intervention as it relates to workflow, structure, and change readiness in implementing EBPIs in the clinic. These providers are also needed in order to assess appropriateness of the intervention for the specific clinic population. NYULH employee participants will be recruited through non-supervisory recruiters, and will be reminded that participation is voluntary and will not affect their employment status.

### Risks and Benefits

Minimal risk to child participants is expected. Assessment visits can result in boredom, performance anxiety, or fatigue. However, most children enjoy study assessment procedures which are designed to be experienced as play; participants may elect to withdraw at any time without penalty, and careful training will be provided to interviewers to recognize when children are refusing to participate in the assessments. Similarly, parents may become bored or distressed as a result of completing questionnaires and interviews about their child’s development. Examiners will be trained to provide appropriate support and follow-up, and personnel supervising assessments will be able to provide referrals should significant concerns about a child’s development arise. In addition, examiners will be trained to provide necessary logistical support to parents during assessments (e.g., if parents cannot read the questionnaires due to literacy levels). As with all studies, there are risks associated with breaches of confidentiality; however, our team will implement all Institutional Review Board (IRB) procedures approved for this study to minimize this risk.

There may be no direct benefits to children participating in this study. However, any positive (atypical) surveillance, screening, or evaluation results with clinical relevance will be discussed with the child’s parents and healthcare provider, and the project will offer (and help facilitate) a referral for an Early Intervention evaluation up to age 3 years as needed. In addition, measures of maternal depression and parenting stress may lead to identification of parents in need of further evaluation and possible treatment; these parents will be offered referral for mental health services.

In addition, minimal risk to NYULH employees is expected. Clinic staff may feel pressure to participate or to answer questions in a specific manner. However, participants will be reminded that all information will be kept confidential. In addition, passive recruitment methods involving a non-supervisory recruiter will be used and the study team will ensure that all participants understand that participation is voluntary and will not impact the status of their employment.

As above, there may be no direct benefits to NYULH employees participating in this study. However, the knowledge related to implementation of integrated programs in the pediatric clinic will have direct effects on how the HealthySteps+PlayReadVIP intervention is delivered, with the potential to improve workflow and patient outcomes.

## Strategies for Recruitment and Retention

**Parent-Child Dyads**

Enrollment will take place at the NYU Langone Sunset Park Family Health Center (SPFHC), a key site that is part of the HealthySteps (HS) networks. A consecutive sample of approximately 500 parent-infant dyads presenting for well-child pediatric appointments, identified through Epic (see Section 4.5.1. or by recommendation of care providers, will be assessed for eligibility

Outreach by Study Team:

1. Trained study team members will provide basic study information to potential parent and provider participants during clinic visits or by phone.
2. If contacted in person, families and providers interested in participation will receive additional information, undergo eligibility screening, and complete informed consent procedures (either electronic or written).
3. If contacted by phone, families interested in participation will receive additional information and then will be met in person at the clinic to undergo eligibility screening and complete informed consent procedures (either electronic or written).

Inclusive Recruitment Approach:

1. The study specifically targets families with higher needs (Tier 3 HealthySteps participants) to ensure the intervention reaches the population most likely to benefit.
2. Both English- and Spanish-speaking families are included, reflecting the linguistic diversity of the population, and all materials will be provided in both languages.

**Care Providers**

Emails will be sent to all pediatric care providers (i.e., pediatricians, residents, nurse practitioners) and providers of HealthySteps and PlayReadVIP programs through mass emails (not targeted at specific individuals) informing them of the study and providing contact information for the study team so that employees can self-identify as interested in the study. A consecutive sample of 25 providers will be recruited by non-supervisory members of the study team through this passive recruitment approach.

As with parent-child dyads, employee participants will be assigned a study ID separate from any personally identifiable information, and all research data will be linked only to the study ID. Study team members and research data will not be used in the performance evaluation or any other evaluations of prospective subjects. We will obtain clearance from the Department of Human Resources before recruiting any NYULH employees.

**Retention Strategies**

*Regular Contact*:

Participants will receive reminder calls, emails, and mailings for clinic visits and assessments. The study team will follow up after missed appointments to encourage continued participation.

*Flexible Scheduling*:

Assessments and follow-ups can be scheduled during evenings and weekends to accommodate participants’ schedules.

*Contact Information*:

Participants will be asked to provide up to two alternate contacts in case they become unreachable.

*Incentives:*

Parent participants will receive up to $60 ($25 per survey and $35 per in-person assessment) per visit for their time and travel expenses. Care providers will receive a team lunch to thank them for their time participating in surveys. This team incentive was recommended by clinic staff as an appropriate reimbursement for care providers.

Both parent and provider participants will receive $40 if they participate in the additional in-depth interview. This amount is comparable to other studies in similar settings and helps to mitigate barriers like transportation costs and time use for employee participants.

*Cultural Competence*:

1) Ethnic minority and community member research assistants and interventionists will be employed whenever possible to build trust.

2) Materials and interventions are tailored to acknowledge and respect parents’ cultural experiences and values.

*Engagement Through Relevance*:

1) The intervention builds on parent goals, incorporating structured processes for self-reflection, goal-setting, and family participation in activities.

2) The SPFHC and its existing relationships with families will provide additional reinforcement to retain participants.

These strategies aim to ensure strong recruitment and retention, allowing the study to meet its objectives of evaluating the effectiveness and implementation of the HS+PlayReadVIP intervention

### Use of DataCore/Epic Information for Recruitment Purposes

This study will utilize EPIC to identify parent-child dyads for recruitment purposes.

Research Coordinators will submit weekly requests to NYULH DataCore via iLab, with up to 75 requests over the course of the study. Reports will be requested for all families with scheduled well-child visits at the NYU Langone Family Health Centers in the upcoming week at the time of request, and who meet inclusion criteria based on child current age, singleton birth, and parent primary language (PHI) and should be approached for recruitment and screening. The PI and Research Coordinators will have access to search results in order to identify potential participants. Research Coordinators will then use the following PHI identifiers to track potential participants in the clinic in order to approach them for recruitment and screening either in the clinic or by phone: name, medical record number (MRN). Data will be discarded at the end of the week for which the DataCore report was requested. All paper records will be shredded using NYULH secure shredding boxes. Electronic records, including emails, will be fully deleted from email and internal servers.

The research team will not have a treating relationship with potential participants. Once potential participants have been identified, the study team will notify the treating physician (TP) in person that they have patients eligible to participate as follow:

- Treating physician (TP) has been notified that the study team will contact potential subjects in-person in the waiting room or by phone.

Once contact is made, approved recruitment language will be used to communicate the reason they are being contacted and potential parent participants will be asked if they are interested in learning more about this specific study. Should the potential participants agree, the study team will provide the individuals with information regarding the next steps for participation.

We will discard identifying information for individuals who do not meet eligiblity criteria and for individuals who do not wish to take part in this study immediately. Deidentified information for all such individuals will be kept until study completion and reported in aggregate for CONSORT and other reporting.

## Informed Consent Process

### Consent/Assent and Other Informational Documents Provided to Participants

Consent forms describing in detail the study intervention, study procedures, and risks are given to the participant and written documentation of informed consent is required prior to starting intervention. The following consent materials are submitted with this protocol:

1. Key Information form (parent): provides all participants with key and concise information about this study.
2. Main consent form (parent): to be signed by all parent participants enrolled in the study, for their own and their child’s participation.
3. Main consent form (provider): to be signed by all employee participants enrolled in the study.
4. Audio/Visual consent form: to be signed by all parent participants enrolled in the study, for themselves and their child, in order to record assessments for later transcription and coding if needed.

IRB-approved English consent materials will be translated into other languages. Translated consent materials will be submitted for IRB review and approval at a future date via a modification submission before use in this study.

### Consent Procedures and Documentation

Informed consent is a process that is initiated prior to the individual’s agreeing to participate in the study and continues throughout the individual’s study participation. Extensive discussion of risks and possible benefits of participation will be provided to the participants and their families. Consent materials will be IRB-approved and trained research staff will read the form with potential participants confidentially.

Parent participants will be approached in the waiting room of the pediatric ambulatory care clinic at NYU Langone Health - Sunset Park Family Health Centers, and will have the opportunity to carefully review the written consent form and ask questions prior to signing. The study staff will explain the research study, including the nature of assessments, inclusion and exclusion criteria, rights and responsibilities of participants, and risks and benefits, and will answer any questions that may arise. All participants will receive a verbal explanation in terms suited to their comprehension.

An IRB-approved and trained study staff will begin the informed consent process with a concise and focused presentation of the key information using an IRB-approved *Key Information Form*. If a potential parent participant is interested in learning more, the study staff will continue describing the study in more details using an IRB-approved main consent form. Participants will have the opportunity to provide consent at the time of the consent discussion. Additionally, participants will be able to take the consent materials home to discuss with family members or others and return to provide consent at another time in-person or remotely, via a phone or video call, to reduce participant burden. E-consenting will be offered through REDCap using the single Institutional Review Board (sIRB) approved informed consent form and sent via text message.

The parent participants will be asked to sign the physical or electronic main consent and AV consent documents prior to any procedures being done specifically for the study. The participants may withdraw consent at any time throughout the course of the study.

A copy of the signed consent documents will be given to the parent participants for their records. The rights and welfare of the participants will be protected by emphasizing to them that the quality of their medical care will not be adversely affected if they decline to participate in this study.

A copy of the signed consent documents will be stored in the participant’s research record. The consent process, including the name of the individual obtaining consent, will be thoroughly documented in the participant’s research record. Any alteration to the standard consent process (e.g. use of a translator, consent from a legally authorized representative, consent document presented orally, etc.) and the justification for such alteration will likewise be documented.

## Duration of Study Participation

Duration of study participation (screening, enrollment, randomization, intervention delivery, and follow-up) for parent-child dyads is approximately 2 years – from infant birth through age 2 follow up, which may take place prior to the child’s 3^RD^ birthday. Participation for care providers is approximately 1 year, with surveys and qualitative interviews taking place to coincide with age 12m and 24m parent-child assessments.

## Total Number of Participants and Sites

Recruitment will end when 500 parent-child dyads and 25 care providers are enrolled. Recruitment and enrollment will take place at NYU Langone Sunset Park Family Health Center (SPFHC).

## Participant Withdrawal or Termination

### Reasons for Withdrawal or Termination

Participants are free to withdraw from participation in the study at any time upon request. An investigator may terminate participation in the study if:

- Any clinical adverse event (AE), laboratory abnormality, or other medical condition or situation occurs such that continued participation in the study would not be in the best interest of the participant
- The participant meets an exclusion criterion (either newly developed or not previously recognized) that precludes further study participation

### Premature Termination or Suspension of Study

This study may be temporarily suspended or prematurely terminated if there is sufficient reasonable cause. Written notification, documenting the reason for study suspension or termination, will be provided by the suspending or terminating party to all applicable authorities. If the study is prematurely terminated or suspended, the PI will promptly inform the IRB and will provide the reason(s) for the termination or suspension.

Circumstances that may warrant termination or suspension include, but are not limited to:

- Determination of unexpected, significant, or unacceptable risk to participants
- Insufficient compliance to protocol requirements

Study may resume once concerns about safety, protocol compliance, data quality are addressed and satisfy the sponsor and/or IRB.

# Behavioral/Social Intervention

## Study Behavioral or Social Intervention(s) Description

The study integrates two evidence-based programs, HealthySteps (HS) and PlayReadVIP, into a combined intervention (HS+PlayReadVIP) to support families and improve early child development (ECD).

HealthySteps (HS):

1) Focuses on addressing Adverse Childhood Experiences (ACEs) and family stressors.

2) Provides parenting guidance, mental health support, and care coordination.

3) Delivered during well-child visits by an HS specialist.

Video Interaction Project (VIP):

1) Promotes Positive Childhood Experiences (PCEs) and strengthens parent-child interactions.

2) Involves recording and reviewing videos of parents engaging with their child to provide feedback and encourage positive practices.

3) Families receive toys, books, and guides to extend activities at home.

Integrated HS+VIP Model:

1) Combines HS and VIP to address both ACEs and PCEs.

2) Delivered during the same pediatric visits for high-risk families, saving time and enhancing impact.

3) Designed to be culturally sensitive and family-centered.

Setting and Delivery:

- Conducted at the NYU Langone Sunset Park Family Health Center, targeting low-income, minority families.
- Delivered over 24 months during routine pediatric visits.

Goals:

- Improve parenting practices and Early Relational Health (ERH).
- Enhance cognitive, language, and social-emotional development in children.
- Reduce ECD disparities by addressing both risks and opportunities within families.

This integrated approach leverages the accessibility of pediatric primary care to maximize its reach and effectiveness.

### Administration of Intervention

The combined HS+PlayReadVIP model is delivered in person or online with 9 structured sessions from infant age 4 months to 36 months, coinciding with pediatric well-child visits. The program is delivered by trained HS Specialists, with a Masters’ degree or higher. Training ensures fidelity, and sessions are tailored to family needs with flexible scheduling. This approach integrates behavioral and social strategies into pediatric care, minimizing disruption to families and providers.

### Procedures for Training Interventionalists and Monitoring Intervention Fidelity

All Interventionists, including child development specialists and PlayReadVIP coaches, will undergo comprehensive training covering theoretical frameworks, developmental screening techniques, video coaching, cultural sensitivity, and communication strategies. Training includes hands-on practice and role-playing. HS Specialists are either clinical social workers with a Masters’ degree or higher, or doctoral-level behavioral health providers. PlayReadVIP coaches are bachelors’ level professionals with a background in early childhood.

To ensure fidelity, interventions will be monitored through session observations, recorded interactions, adherence checklists, periodic refresher training, and feedback meetings. All training and monitoring activities will be documented, including interventionist competency assessments and fidelity evaluations, to maintain consistent delivery according to protocol.

# Study Procedures and Schedule

## Study Procedures/Evaluations

**Overall Study Procedures:**

For all parent/child dyads who are enrolled in the RCT, we will administer assessments at baseline, 12 months, and 2 years to measure outcomes (e.g., parenting assets/vulnerabilities, parenting practices/parent-child relationship, child development) and covariates (e.g., socio-demographics, biological risk) for Specific Aims 1 and 2. Assessments will include a combination of parent survey, observation of parent/child, direct assessment of child, and medical record review. In addition, we will conduct two assessments with care providers within the clinic to assess intervention implementation. All assessments will be completed by highly trained research assistants blind to group/intervention status.

### Study Specific Procedures

**Randomization Procedures**

We will use simple randomization with a maximum tolerated imbalance procedure. Before randomization to HS+PlayReadVIP or standard HS, enrolled families will complete baseline assessments. Families will receive HS+PlayReadVIP or standard HS at each well-child visit through 24M.

**Assessment Procedures**

Assessments will be conducted at 12M and 24M (critical transition points for both parenting and child development) by highly trained, bilingual research staff, masked to intervention condition, to reduce bias. Assessments will last ~1 hour, in line with prior studies of parents of young children. Assessments will include surveys/questionnaires, observations of parents and children, and direct child assessments.

Outcomes to be assessed via questionnaire include:

|  | | | | | |
| --- | --- | --- | --- | --- | --- |
| **Level** | **Construct** | **Instrument** | **Base-line** | **12M** | **24M** |
| Effectiveness Outcomes | | | | | |
| Parent | **Parent-Child Relationship Quality** | Adult-Child Relationship Scale (*α*=.69-.84)^142^ |  | x | x |
|  | **Responsivity/Cognitive Stimulation** | StimQ Reading, Teaching/Play, Verbal Responsivity (*α*=.88)^143^ |  | x | x |
|  | Stress/Mental Health | Edinburgh Depression Scale (*α*=.77-.86)^144^, Perceived Stress Scale ( *α*=.75-.91);^145^ | x | x | x |
|  | Self-Efficacy | Parenting Self-Agency Measure (*α*=.68-.7)^146^ | x | x | x |
|  | Social Support | General Life Satisfaction (*α*=.91),^147^ Dyadic Adjustment Scale (*α*=.7-.95)^148^ | x | x | x |
|  | Structure | limit-setting, proactive parenting, feeding/sleep/screentime routines,^149^ Socolar Discipline Survey (*α*=.56-.82)^150^ |  | x | x |
|  |  | HealthySteps Parent Feedback Survey^170^ |  | x | x |
|  |  | Parenting Daily Hassles^171^ |  | x | x |
| Child | **Social Competence** | Infant Behavior Questionnaire (*α*=.75-.78),^151^ Brief Infant Toddler Social-Emotional Assessment (*α*=.69-.8)^152^ |  | x |  |
|  |  | Child Behavior Checklist (α=.9-.94)^153^ |  |  | x |
|  | Language & Cognitive Development | Early Social Communication Scale (*α*=.61-.91),^154^  MacArthur-Bates Communicative Development Inventories (*α*=.86);^155^  Dual Language Learners English-Spanish Inventories^172^ |  | x | x |
| Implementation Outcomes (CFIR, RE-AIM, CIHS) | | | | | |
| EBPI Provider (care-team member) | Acceptability | Acceptability of Intervention Measure (AIM; *α*=.89)^158^ |  | x | x |
|  | Appropriateness | Intervention Appropriateness Measure (IAM; *α*=.87)^158^ |  | x | x |
|  | CFIR Domains | Evidence-based Practice Attitudes Scale-36 (*α*=.80),^159^ clinic culture, perceived complexity of intervention, external policies, competence | x | x | x |
|  | Acceptability | Parent satisfaction surveys^117^ |  | x | x |
|  | | | | | |

In addition, we will assess parent-child relationship quality through observation of videotaped interactions between parents and children at 12 months and 2 years, including play, shared reading, and teaching activities, with global coding of parent and child interaction quality (e.g., responsiveness, sensitivity, positive/negative regard).^138-141^

Direct assessment of child language development using the Receptive and Expressive One-Word Picture Vocabulary Tests^156,157^ will be conducted at the 2 year assessment.

Finally, qualitative interviews will be conducted to examine implementation outcomes, including:

| **Level** | **Domains** | **Measure** | **Base-line** | **12M** | **24M** |
| --- | --- | --- | --- | --- | --- |
| EBPI Provider (care-team member) | Acceptability | ease of program delivery, confidence/efficacy of program delivery |  |  | x |
|  | Appropriateness | perception that program meets needs of patient population |  |  | x |
|  | Fidelity | adherence and perceptions of program delivery |  |  | x |
|  | CFIR Domains | clinic culture, perceived complexity of intervention, external policies, competence |  |  | x |
|  | Acceptability | Parent satisfaction with intervention components |  |  | x |
| Parent | Appropriateness | perceptions that needs are met, content/coverage/frequency/ duration of program sessions, “essential” components |  |  | x |
|  | CFIR Domains | home environment, family needs, resources, motivation |  |  | x |
|  | CFIR Domains | home environment, family needs, resources, motivation |  |  | x |

### Standard of Care Study Procedures

All families will receive all routine pediatric care, and families enrolled in the Control group will receive standard HealthySteps.

### Care Provider Study Procedures

Directly after consent, at the time of initial implementation of the HealthySteps+PlayReadVIP program—that is, enrollment and baseline assessment of first participants—providers will be asked to complete the baseline survey. Then, at 6 and 18 months following consent and initial implementation of HealthySteps+PlayReadVIP, enrolled care providers will be asked to complete online surveys related to implementation outcomes, including appropriateness of the intervention, and acceptability for both parent participants and providers. These assessments are equivalent in timing to the first 12 and 24 month parent-child assessments. In addition, prior to initial implementation and at the two following assessment points, providers will be asked to complete surveys related to attitudes towards evidence-based programs, clinic climate, and their feelings of competency and understanding of the HealthySteps+PlayReadVIP program. Baseline assessments prior to any implementation of the HealthySteps+PlayReadVIP program are expected to take approximately 15 minutes, and assessments at 6 and 18 months following initial implementation will take up to 30 minutes. Providers will be able to access and complete the surveys at a time convenient to them, and will be encouraged to complete surveys outside of their normal work hours in order to reduce bias and maintain confidentiality.

In addition to these surveys, qualitative interviews will be used to gain an in depth understanding of barriers and perceptions to implementation, building on the information from the quantitative surveys.

## Study Schedule

### Screening

**Screening Visit (Day -3 to -1)**

- Review medical history to determine eligibility based on inclusion/exclusion criteria.
- Confirm eligibility in person with potential participants based on inclusion/ exclusion criteria, including primary language (i.e., administer the language screener when English or Spanish is not the primary language), ability to provide consent (i.e., test participant’s comprehension of study procedures and components), and viable contact methods (phone or email) for future participation in the study.

### Enrollment/Baseline

**Enrollment/Baseline Visit (Visit 1, Day 0)**

- Obtain informed consent of potential participant verified by signature on study informed consent form.
- Verify inclusion/exclusion criteria.
- Obtain demographic information, baseline information regarding parent health, mental health, the home environment, social determinants of health, and children’s development.

### Intermediate Visits

**Visit 2 (Day 180 +/- 90)**

- Record adverse events as reported by participant or observed by investigator.
- Administer questionnaires/surveys of parent well-being, parenting, and child development, and conduct parent-child observations
- Administer questionnaires to parents and care providers regarding program acceptability, appropriateness, and reach.
- Record participant’s engagement to intervention program and program fidelity and reach.

### Final Study Visit

***Final Study Visit (Visit 3, Day 540 +/- 90)***

- Record adverse events as reported by participant or observed by investigator.
- Administer questionnaires/surveys of parent well-being, parenting, and child development, and conduct parent-child observations
- Administer questionnaires to parents and care providers regarding program acceptability, appropriateness, and reach.
- Record participant’s engagement to intervention program and program fidelity and reach.
- Conduct qualitative interviews regarding program implementation with parents and care providers.

# Assessment of Safety

## Specification of Safety Parameters

This is a study behavioral and social aspects of parent-child interactions, promoting healthy development and reducing disparities in pediatric care. Minimal risk to participants is expected. There are no specific Adverse Events (AE) or Serious Adverse Events (SAE) related to study participation of which we are aware that can be specified in advance of the study.

### Definition of Adverse Events (AE)

An ***adverse event*** (AE) is any symptom, sign, illness or experience that develops or worsens in severity during the course of the study. Intercurrent illnesses or injuries should be regarded as adverse events. Abnormal results of diagnostic procedures are considered to be adverse events if the abnormality:

- results in study withdrawal
- is associated with a serious adverse event
- is associated with clinical signs or symptoms
- leads to additional treatment or to further diagnostic tests
- is considered by the investigator to be of clinical significance

This is a minimal risk study; any potential risks are described earlier (in ‘Risks’ section above). There are no AEs related to study participation that can be defined or specified in advance of the study.

### Definition of Serious Adverse Events (SAE)

**Serious Adverse Event**

Adverse events are classified as serious or non-serious. A ***serious adverse event*** is any AE that is:

- fatal
- life-threatening
- requires or prolongs hospital stay
- results in persistent or significant disability or incapacity
- a congenital anomaly or birth defect
- an important medical event

Important medical events are those that may not be immediately life threatening, but are clearly of major clinical significance. They may jeopardize the subject, and may require intervention to prevent one of the other serious outcomes noted above. For example, drug overdose or abuse, a seizure that did not result in in-patient hospitalization, or intensive treatment of bronchospasm in an emergency department would typically be considered serious.

All adverse events that do not meet any of the criteria for serious should be regarded as ***non-serious adverse events***.

### Definition of Unanticipated Problems (UP)

**Unanticipated Problems Involving Risk to Subjects or Others**

Any incident, experience, or outcome that meets all of the following criteria:

- Unexpected in nature, severity, or frequency (i.e. not described in study-related documents such as the IRB-approved protocol or consent form, the investigators brochure, etc)
- Related or possibly related to participation in the research (i.e. possibly related means there is a reasonable possibility that the incident experience, or outcome may have been caused by the procedures involved in the research)
- Suggests that the research places subjects or others at greater risk of harm (including physical, psychological, economic, or social harm).

## Classification of an Adverse Event

### Severity of Event

For AEs not included in the protocol defined grading system, the following guidelines will be used to describe severity:

- **Mild** – Events require minimal or no treatment and do not interfere with the participant’s daily activities.
- **Moderate** – Events result in a low level of inconvenience or concern with the therapeutic measures. Moderate events may cause some interference with functioning.
- **Severe** – Events interrupt a participant’s usual daily activity and may require systemic drug therapy or other treatment. Severe events are usually potentially life-threatening or incapacitating.

### Relationship to Study Intervention

*The clinician’s assessment of an AE's relationship to study intervention is part of the documentation process, but it is not a factor in determining what is or is not reported in the study. If there is any doubt as to whether a clinical observation is an AE, the event should be reported. All AEs must have their relationship to study intervention assessed. In a clinical trial, the study intervention must always be suspect. To help assess, the following guidelines are used:*

- ***Related*** *– The AE is known to occur with the study intervention, there is a reasonable possibility that the study intervention caused the AE, or there is a temporal relationship between the study intervention and event. Reasonable possibility means that there is evidence to suggest a causal relationship between the study intervention and the AE.*
- ***Not Related*** *– There is not a reasonable possibility that the administration of the study intervention caused the event, there is no temporal relationship between the study intervention and event onset, or an alternate etiology has been established.*

### Expectedness

This is a minimal risk study; any potential risks are described earlier (in ‘Risks’ section above). There are no expected AEs or SAEs.

## Time Period and Frequency for Event Assessment and Follow-Up

The occurrence of an AE or SAE may come to the attention of study personnel during study visits and interviews of a study participant presenting for medical care, or upon review by a study monitor. All AEs including local and systemic reactions not meeting the criteria for SAEs will be captured on the appropriate RF. Information to be collected includes event description, time of onset, clinician’s assessment of severity, relationship to study intervention (assessed only by those with the training and authority to make a diagnosis), and time of resolution/stabilization of the event. All AEs occurring while on study must be documented appropriately regardless of relationship. All AEs will be followed to adequate resolution.

Any medical condition that is present at the time that the participant is screened will be considered as baseline and not reported as an AE. However, if the study participant’s condition deteriorates at any time during the study, it will be recorded as an AE. UPs will be recorded in the data collection system throughout the study.

Changes in the severity of an AE will be documented to allow an assessment of the duration of the event at each level of severity to be performed. AEs characterized as intermittent require documentation of onset and duration of each episode.

The PI will record all reportable events with start dates occurring any time after informed consent is obtained until 7 (for non-serious AEs) or 30 days (for SAEs) after the last day of study participation. At each study visit, the investigator will inquire about the occurrence of AE/SAEs since the last visit. Events will be followed for outcome information until resolution or stabilization.

All unresolved adverse events should be followed by the investigator until the events are resolved, the subject is lost to follow-up, or the adverse event is otherwise explained. At the last scheduled visit, the investigator should instruct each subject to report any subsequent event(s) that the subject, or the subject’s personal physician, believes might reasonably be related to participation in this study. The investigator should notify the study sponsor of any death or adverse event occurring at any time after a subject has discontinued or terminated study participation that may reasonably be related to this study.

## Reporting Procedures – Notifying the IRB

### Adverse Event Reporting

At each contact with the subject, the investigator must seek information on adverse events by specific questioning and, as appropriate, by examination. Information on all adverse events should be recorded immediately in the source document, and also in the appropriate adverse event module of the case report form (CRF). All clearly related signs, symptoms, and abnormal diagnostic procedures results should recorded in the source document, though should be grouped under one diagnosis.

All adverse events occurring during the study period must be recorded. The clinical course of each event should be followed until resolution, stabilization, or until it has been determined that the study treatment or participation is not the cause. Serious adverse events that are still ongoing at the end of the study period must be followed up to determine the final outcome. Any serious adverse event that occurs after the study period and is considered to be possibly related to study participation should be recorded and reported immediately.

### Serious Adverse Event Reporting

If the report is supplied as a narrative, the minimum necessary information to be provided at the time of the initial report includes:

| - Study identifier - Study Center - Subject number - A description of the event - Date of onset | - Current status - Whether study treatment was discontinued - The reason why the event is classified as serious - Investigator assessment of the association between the event and study treatment |
| --- | --- |

### Unanticipated Problem Reporting

Incidents or events that meet the OHRP criteria for UPs require the creation and completion of an UP report form. It is the site investigator’s responsibility to report UPs to their IRB and to the DCC/study sponsor. The UP report will include the following information:

- Protocol identifying information: protocol title and number, PI’s name, and the IRB project number;
- A detailed description of the event, incident, experience, or outcome;
- An explanation of the basis for determining that the event, incident, experience, or outcome represents an UP;
- A description of any changes to the protocol or other corrective actions that have been taken or are proposed in response to the UP.

To satisfy the requirement for prompt reporting, UPs will be reported using the following timeline:

- UPs that are SAEs will be reported to the IRB and to the study sponsor within 24-72 hours of the investigator becoming aware of the event.
- Any other UP will be reported to the IRB and to the study sponsor within 2 weeks of the investigator becoming aware of the problem.
- All UPs should be reported to appropriate institutional officials (as required by an institution’s written reporting procedures), the supporting agency head (or designee), and OHRP within 2 weeks of the IR’s receipt of the report of the problem from the investigator.

## Reporting Procedures – Notifying the Study Sponsor

The study clinician will complete a SAE Form within the following timelines:

- All deaths and immediately life-threatening events, whether related or unrelated, will be recorded on the SAE Form and submitted to the DCC/study sponsor within 24 hours of site awareness. See Section 1, Key Roles for contact information.
- Other SAEs regardless of relationship will be submitted to the DCC/study sponsor within 72 hours of site awareness.

All SAEs will be followed until satisfactory resolution or until the site investigator deems the event to be chronic or the adherence to be stable. Other supporting documentation of the event may be requested by the DCC/study sponsor and should be provided as soon as possible.

As a follow-up to the initial report, within the following 48 hours of awareness of the event, the investigator shall provide further information, as applicable, on the unanticipated event or the unanticipated problem in the form of a written narrative. This should include a copy of the completed Unanticipated Problem form, and any other diagnostic information that will assist the understanding of the event. Significant new information on ongoing unanticipated adverse effects shall be provided promptly to the study sponsor.

## Safety Oversight

It is the responsibility of the Principal Investigator to oversee the safety of the study at their site. This safety monitoring will include careful assessment and appropriate reporting of adverse events as noted above, as well as the construction and implementation of a site data safety-monitoring plan. Medical monitoring will include a regular assessment of the number and type of serious adverse events.

The project will establish a Data Safety Monitoring Plan (DSMP) in which adverse events (AEs), serious adverse events (SAEs), and unanticipated problems (UPs) related to participation in the study, if identified, will be documented through a centralized database system, investigated, and followed up. The Principal Investigator (PI), Sub-Investigators (Sub-Is), and Project Manager will be well-versed in the reporting procedures described below. All investigators and staff involved in this project have completed an extensive course and passed a certifying exam on the protection of human subjects in research.

As this is a non-invasive, minimal risk study of a preventive intervention, no predefined stopping rules will be used.

To further protect participants and ensure the collection of high-quality data, a Data Safety Monitoring Board (DSMB) will be established for this proposal. The DSMB will be responsible for monitoring participant safety, monitoring data quality, and evaluating the progress of the study, including reviewing the research protocol and informed consent documents, assessing participant risk versus benefit, reporting to the NICHD as appropriate on the safety and progress of the trial, and making recommendations and resolutions of problems reported by the PI. Members of the DSMB will consist of experts or representatives in the fields of relevant clinical expertise. Members will not have any conflict of interest pertaining to this study. DSMB reports will be submitted to the IRB according to IRB policies and procedures. The members of the DSMB will be determined after the outcome of the grant application has been decided.

***Data Safety Monitoring Board***

Membership and Affiliation

The DSMB will consist of a minimum of three members (including the DSMB Chair) and include, but is not limited to, representatives from pediatrics, clinical trials methodology, and/or other applicable fields. Members will serve on the DSMB for a two-year term and will remain eligible for reappointment upon their term’s conclusion.

Conflicts of Interest

Each DSMB member will sign a Conflict of Interest Statement which includes any other relationship that could be perceived as a conflict of interest related to the study and/or associated with commercial interests pertinent to study objectives.

DSMB Responsibilities

Prior to initiation of the study, a detailed DSMB charter will be created. The DSMB charter will be submitted to the IRB prior to the enrollment of the first subject. The DSMB charter will provide a detailed list of the DSMB responsibilities. These responsibilities include, but are not limited to:

Review the research protocol, informed consent documents and plans for data safety and monitoring;

Recommend subject recruitment be initiated after receipt of a satisfactory protocol;

Evaluate the progress of the trial, including periodic assessments of data quality and timeliness, recruitment, accrual and retention, participant risk versus benefit, performance of the trial sites, and other factors that can affect study outcome;

Consider factors external to the study when relevant information becomes available, such as scientific or therapeutic developments that may have an impact on the safety of the participants or the ethics of the trial;

Review study performance, make recommendations and assist in the resolution of problems reported by the Principal Investigator;

Protect the safety of the study participants, including from the main potential risks of the study:

Violation/loss of confidentiality

Discomfort due to disclosure of sensitive information

Concern regarding identification of child abuse/neglect, domestic violence, or other criminality

Report to IRB and/or NICHD on the safety and progress of the trial;

Make recommendations to the IRB, NICHD, and/or the PI concerning continuation, termination or other modifications of the trial based on the observed beneficial or adverse effects of the treatment under study;

Ensure the confidentiality of the study data and the results of monitoring; and,

Assist the NICHD by commenting on any problems with study conduct, enrollment, sample size, and/or data collection.

Protection of Confidentiality

Investigators will expunge all patient, informant, and provider-level identifiers from the dataset prior to providing it to DSMB members during meetings or in reports. All data and discussions conducted by the DSMB will remain confidential. Participant identities will remain concealed from DSMB members.

Content of DSMB Reports

The study PI will ensure that the DSMB is apprised of all new safety and risk information relevant to the study, and of all protocol amendments. Summary safety data, enrollment data, and other progress reports (e.g. protocol deviations, site monitoring summaries) will be provided to the DSMB prior to its annual meeting and at other times as needed.

In the event of a related SAE, a report containing the study title and number, subject’s ID number, date of signed consent, SAE and date of event, narrative explanation (e.g., subject’s major complaint, brief description of event’s evaluation and disposition, pertinent mental health or behavioral assessments, intervention start date, time of last participation, any prior SAE, the PI’s assessment whether the event was expected, and its relatedness to the study’s intervention) will be provided to the DSMB within 48 hours.

Frequency of Meetings

The DSMB will meet in-person or through teleconference a minimum of once annually to review data related to enrollment progress and trial implementation, and to evaluate the ongoing safety of study subjects. Meetings may be cancelled by the Chair if no clinical study review is required. Additional DSMB meetings may be held if it is determined that more frequent study review is required. A detailed agenda will be provided for each meeting. Reports will be provided to DSMB members via secure methods. Based on these reviews, the DSMB will make recommendations to the PI and/or the NICHD Project Officer concerning the continuation, modification, or termination of the trial. The roles and responsibilities of committee members and meeting procedures will be formally described in a charter.

# Statistical Considerations

## Statistical and Analytical Plans

***Effectiveness Outcomes***

Initial ANOVA and Chi-square tests will be conducted to examine baseline equivalence of randomly assigned groups. Analyses with sex as a biological variable, including moderation and subgroup analyses, will be performed. Covariate entry (e.g., sociodemographics) will be decided based on model fit criteria, and sensitivity analyses will be performed for post-randomization risks.

Missing data and attrition: The research team has expertise in minimizing missing data. In addition, multiple imputation (MI)^160^ will be used to replace item-level data using the rich array of concurrently collected variables and prior responses. If an entire measure/survey is missing, we will use chained equations with lagged (in time) variables^161^ using all available information to build a large set of imputed datasets. Estimates will be combined using Rubin’s rules.^160^ When data are Missing at Random, likelihood-based methods provide unbiased estimates of population parameters even when panels are incomplete. In addition, we will use attrition analyses^162^ to examine whether attrition is related to baseline characteristics.

Tests of hypotheses: In order to examine the added value of HS+VIP in comparison to the standard HS model, we will focus on outcomes related to ERH and ECD where we expect the effects of standard HS to be substantially smaller than the integrated HS+VIP model. Impact analyses at each assessment timepoint will be conducted using ordinary least squares (OLS) regression in an intent-to-treat (ITT) framework, adjusted for baseline sociodemographic covariates. In addition, structural equation models (SEM)^163^ will be used to examine family outcomes over time. This will allow us to examine whether initial levels and trajectories in parent well-being, parent-child interaction, and ECD vary between participants, and whether that variation is explained by assignment to HS+VIP or standard HS.

***Implementation Outcomes***

Initial analyses will include examination of measure distribution, tests of normality, and confirmatory factor analysis, to create multi-indicator, multi-informant constructs where appropriate. Reach: Effect sizes of differences between 1) the study sample and clinic population, and 2) those who were eligible and enrolled vs. those who were eligible and declined will be determined by descriptive comparisons of multiple sociodemographic characteristics, including race/ethnicity, maternal age, child gender, and income. Fidelity: Summary scores will be created for fidelity data across sessions (i.e., computing proportion of HS and/or VIP session components completed on average, and the interquartile range). Scores will be computed separately for VIP (PCEs related) and HS (ACEs related) content for component analysis to understand whether differential implementation outcomes/challenges occur when HS+VIP is implemented jointly. Engagement: Chi-square tests for independence will be used to examine differences between the proportion of visits attended in the HS+VIP and standard HS models.

Acceptability: To investigate acceptability among parent participants, SEM will be used to examine AIM scores across assessment timepoints and to examine whether trajectories in parent AIM scores vary by participation or sociodemographic characteristics. In addition, multivariate ANOVA will be used to examine acceptability across interested parties at different levels (parents, HS+VIP care team). Finally, confirmatory factor analysis will be used to create a multi-informant acceptability construct and multivariate ANOVA will again be used to examine differences in overall acceptability across HS+VIP and standard HS groups.

Appropriateness: Analysis of appropriateness will mirror analyses used to examine acceptability.

***Mediation Through Intervention/Health Disparities Theory of Change***

In order to examine whether changes in ECD outcomes at 24M result from program impacts on parents and ERH at 12M, mediation analysis will be used. Analyses will be conducted according to principles set forth by MacKinnon^164^ and Hayes,^165^ using SEM with bias-corrected bootstrapping. Bootstrapping provides an estimate of the sampling distribution of the indirect effect by resampling from the obtained sample with replacement and estimating the path coefficients for each resample. The confidence interval for the indirect effect is calculated from percentile-based lower and upper bounds in the ordered list of the sampling distribution. Confidence interval bounds are bias-corrected to account for skew common in human subjects data.^165^ Mediators will be examined separately, and only mediators on which HS+VIP has a significant impact at 12M will be tested.

***Moderation of Effectiveness Outcomes by Implementation Factors***

Moderation analysis using OLS regression will be used to determine whether program impacts vary based on CFIR contexts. Regression models will be built on those in Aim 2. Multiplicative interaction terms between group assignment and individual moderators will be added.

***Moderation of Implementation Outcomes***

To examine how CFIR individual and intervention-related factors predict implementation fidelity, multivariate regression analysis will be used. We will regress fidelity outcomes (fidelity in delivery, receipt, and enactment) on parents’ perception about the intervention, family characteristics, and child characteristics. Three fidelity outcomes will be examined in separate analyses.

***Qualitative Analysis***

Transcripts will be reviewed for emerging themes using an iterative process of textual analysis. The constant comparative method will be used to identify and refine codes and devise a final code structure.^134^ Transcripts will initially be read in their entirety to gain insight into broad themes and to note repeating themes. Themes and subthemes will be reviewed to confirm consistency, coherence, and distinctiveness before defining and finalizing a codebook that reflects the data’s meaning. Each reader will do multiple passes to get the full picture of discussion flow and consensus will be reached for each quotation. Data organization and retrieval will be facilitated by Dedoose software.

***Data Synthesis***

Through triangulation, a process for strategically utilizing both methods together, we will examine convergence and expansion.^135^ Convergence assesses whether quantitative and qualitative results provide the same answer to the same question (e.g., does quantitative assessment of barriers concur with qualitative data around barriers/facilitators of implementation). Expansion assesses whether the qualitative data can explain unanticipated findings produced by quantitative data (e.g., can quantitative data that suggests little variation in clinic structures across different types of clinics be further explained by qualitative data).

## Statistical Hypotheses

***Effectiveness***

*Null Hypothesis (H0):* The integrated HS+PlayReadVIP intervention does not significantly improve parenting practices and relational health outcomes compared to HS alone.

*Alternative Hypothesis (H1)*: The integrated HS+PlayReadVIP intervention significantly improves parenting practices and relational health outcomes compared to HS alone.

***Implementation***

*Null Hypothesis (H0)*: The HS+PlayReadVIP intervention is not feasible, acceptable, appropriate, or scalable in real-world pediatric primary care settings.

*Alternative Hypothesis (H1)*: The HS+PlayReadVIP intervention is feasible, acceptable, appropriate, and scalable in real-world pediatric primary care settings.

## Analysis Datasets

The project will produce data obtained from surveys/questionnaires, interviews, direct assessments, and observations. Data will be collected from ~500 parent-child dyads, generating several raw datasets. Data will be collected and stored in secure REDCap databases. Datasets will be merged for analysis and stored in CSV format. We will produce raw data files to be transformed into final datasets for statistical analysis at the individual level.

## Description of Statistical Methods

For general and primary endpoint analytic methods, see above.

### Safety Analyses

Summary statistics for AEs and SAEs will be examined. AEs will be coded according the severity (see Section 8.2.1), and the number, severity, frequency, and relation to the study will be presented to the DSMB and other audiences of interest.

### Adherence and Retention Analyses

Participant engagemet in the intervention (e.g., number of possible sessions attended) and fidelity of intervention delivery (e.g., number of program components delivered at each session) will be tracked from the medical record and presented using summary statistics. As noted above, missing data and attrition analyses will be conducted to determine if data is missing at random or missing completely at random, and multiple imputation will be used to account for missing data.

### Baseline Descriptive Statistics

Intervention and control groups will be compared at baseline for differences in sociodemographic characteristics, including child gender, parity, maternal education, income, and mental health, using omnibus F-tests, t-tests, and chi-square tests.

### Planned Interim Analysis

#### Efficacy Review

The same analyses described above will be conducted after assessments at 12 months to examine efficacy outcomes after 6-12 months of intervention delivery. This analysis will not impact final efficacy analyses, although we will address the potential higher rate of type I error due to multiple comparisons (multiple parent and child characteristics), we will use the false discovery rate approach.

### Multiple Comparison/Multiplicity

As noted above, we will use the false discovery rate approach to correct for the potential higher rate of Type I error due to multiple comparisons.

### Tabulation of Individual Response Data

Individual participant data will be listed by measure and time point, and datasets will be available in both wide and long form.

## Sample Size

Sample size was estimated based on a cumulative attrition rate of 15-20% by 24M, similar to previous studies of PlayReadVIP in New York City, and a conservative effect size of HS+PlayReadVIP for main impact analyses of *d* = .30. This effect size is below previous studies of PlayReadVIP alone (*d* = .3-.5), accounting for the potential for non-additive impacts of HS+PlayReadVIP following integration with standard HS. With enrollment of 500 families, we expect a final sample size of 400, which provides more than 80% power to detect an effect size of *d* = ~.3 given alpha = .05 in ANOVA analyses,^166^ a small-to-medium effect size of *f^2^*=.04 in our most complex OLS regression analyses including moderation, and β*_YX_* = 0.3, with only moderate item reliability^167^ in SEM analyses. Our final sample of 400 will provide 80% power to detect a small-to-medium effect size (.26) in mediation analyses based on previous simulation studies.^168^ Analysis of fidelity of delivery will be based on number of sessions (~1125-1500, or 7-10 sessions per family over 2 years), rather than number of families. We have 80% power to detect a small effect size of β*_YX_* = 0.1 in these SEM models.^167^

## Measures to Minimize Bias

### Enrollment/Randomization/Masking Procedures

***Enrollment Procedures:***

Consecutive enrollment of eligible participants, based on screening of inclusion/exclusion criteria by study staff, will take place at the SPFHC pediatric clinic. Baseline assessments, including demographic data and initial surveys/questionnaires, will be conducted at the time of enrollment, after informed consent is collected but before randomization.

***Randomization Procedures***:

Participants will be randomized into intervention (HS+PlayReadVIP) or control (standard HS) groups using a simple randomization with maximum tolderated imbalance procedure. Randomization will be conducted using a secure, centralized randomization system to ensure allocation concealment, and will be conducted by the HS+PlayReadVIP provider to ensure blinding of research staff. Randomization numbers (1 or 2) will be generated in the statistical program R using big stick design with a maximum difference between groups of 4. Numbers will be uploaded to REDCap and Research Coordinators will utilize the REDCap randomization instrument to randomly assign participants to groups based on the generated random number list.

***Replacement of Participants***:

Replacement of participants who discontinue early will not be conducted.

***Masking Procedures***:

Masking will be applied to all research staff conducting data collection assessments. Blinded research staff will not be given access to databases or materials that have the potential to reveal intervention group. Further, all databases and materials with intervention information will be clearly marked UNBLINDING, to protect against inadvertent unmasking.

Interventionists and participants cannot be masked due to the nature of the intervention.

# Source Documents and Access to Source Data/Documents

Source data is all information, original records of clinical findings, observations, or other activities in a clinical trial necessary for the reconstruction and evaluation of the trial. Source data are contained in source documents. Examples of these original documents, and data records include: hospital records, clinical and office charts, laboratory notes, memoranda, subjects’ diaries or evaluation checklists, pharmacy dispensing records, recorded data from automated instruments, copies or transcriptions certified after verification as being accurate and complete, microfiches, photographic negatives, microfilm or magnetic media, x-rays, subject files, and records kept at the pharmacy, at the laboratories, and at medico-technical departments involved in the clinical trial.

The study case report form (CRF) is the primary data collection instrument for the study. All data requested on the CRF must be recorded. All missing data must be explained. If a space on the CRF is left blank because the procedure was not done or the question was not asked, write “N/D”. If the item is not applicable to the individual case, write “N/A”. All entries should be printed legibly in black ink. If any entry error has been made, to correct such an error, draw a single straight line through the incorrect entry and enter the correct data above it. All such changes must be initialed and dated. DO NOT ERASE OR WHITE OUT ERRORS. For clarification of illegible or uncertain entries, print the clarification above the item, then initial and date it.

Access to study records will be limited to IRB-approved members of the study team. The investigator will permit study-related monitoring, audits, and inspections by the IRB/EC, the sponsor, government regulatory bodies, and University compliance and quality assurance groups of all study related documents (e.g. source documents, regulatory documents, data collection instruments, study data etc.). The investigator will ensure the capability for inspections of applicable study-related facilities (e.g. pharmacy, diagnostic laboratory, etc.).

Participation as an investigator in this study implies acceptance of potential inspection by government regulatory authorities and applicable University compliance and quality assurance offices.

# Quality Assurance and Quality Control

QC procedures will be implemented beginning with the data entry system and data QC checks that will be run on the database will be generated. Any missing data or data anomalies will be communicated to the site(s) for clarification/resolution.

Following written SOPs, the monitors will verify that the clinical trial is conducted and data are generated, documented (recorded), and reported in compliance with the protocol, GCP, and the applicable regulatory requirements (e.g., Good Laboratory Practices (GLP), Good Manufacturing Practices (GMP)).

The investigational site will provide direct access to all trial related sites, source data/documents, and reports for the purpose of monitoring and auditing by the sponsor, and inspection by local and regulatory authorities.

# Ethics/Protection of Human Subjects

## Ethical Standard

The investigator will ensure that this study is conducted in full conformity with Regulations for the Protection of Human Subjects of Research codified in 45 CFR Part 46.

## Institutional Review Board

The protocol, informed consent form(s), recruitment materials, and all participant materials will be submitted to the IRB for review and approval. Approval of both the protocol and the consent form must be obtained before any participant is enrolled. Any amendment to the protocol will require review and approval by the IRB before the changes are implemented to the study. All changes to the consent form will be IRB approved; a determination will be made regarding whether previously consented participants need to be re-consented.

## Participant and Data Confidentiality

Information about study subjects will be kept confidential and managed according to the requirements of the Health Insurance Portability and Accountability Act of 1996 (HIPAA). Those regulations require a signed subject authorization informing the subject of the following:

- What protected health information (PHI) will be collected from subjects in this study
- Who will have access to that information and why
- Who will use or disclose that information
- The rights of a research subject to revoke their authorization for use of their PHI.

In the event that a subject revokes authorization to collect or use PHI, the investigator, by regulation, retains the ability to use all information collected prior to the revocation of subject authorization. For subjects that have revoked authorization to collect or use PHI, attempts should be made to obtain permission to collect at least vital status (i.e. that the subject is alive) at the end of their scheduled study period.

Participant confidentiality is strictly held in trust by the participating investigators, their staff, and the sponsor(s) and their agents. This confidentiality is extended to cover testing of clinical information relating to participants. Therefore, the study protocol, documentation, data, and all other information generated will be held in strict confidence. No information concerning the study or the data will be released to any unauthorized third party without prior written approval of the sponsor.

The study monitor, other authorized representatives of the sponsor, or representatives of the IRB may inspect all documents and records required to be maintained by the investigator, including but not limited to, medical records (office, clinic, or hospital) and pharmacy records for the participants in this study. The clinical study site will permit access to such records.

The study participant’s contact information will be securely stored at each clinical site for internal use during the study. At the end of the study, all records will continue to be kept in a secure location for as long a period as dictated by local IRB and Institutional regulations.

Study participant research data, which is for purposes of statistical analysis and scientific reporting, will be transmitted to and stored at NYU Grossman School of Medicine. This will not include the participant’s contact or identifying information. Rather, individual participants and their research data will be identified by a unique study identification number. The study data entry and study management systems used by clinical sites and by NYU Grossman School of Medicine research staff will be secured and password protected. At the end of the study, all study databases will be de-identified and archived at the at NYU Grossman School of Medicine and through the NIH/NICHD Data and Specimen Hub (DASH).

To further protect the privacy of study participants, a Certificate of Confidentiality will be obtained from the NIH. This certificate protects identifiable research information from forced disclosure. It allows the investigator and others who have access to research records to refuse to disclose identifying information on research participation in any civil, criminal, administrative, legislative, or other proceeding, whether at the federal, state, or local level. By protecting researchers and institutions from being compelled to disclose information that would identify research participants, Certificates of Confidentiality help achieve the research objectives and promote participation in studies by helping assure confidentiality and privacy to participants.

### Research Use of Data

Intended Use: Data collected under this protocol may be used to study parenting and child development. No genetic testing will be performed.

Storage: Data will be stored using codes assigned by the investigators. Data will be kept in password-protected computers. Only investigators will have access to the data.

Tracking: Data will be tracked using protected databases in REDCap.

## Secondary Use of Stored Data

Data collected for this study will be analyzed and stored at NYU Grossman School of Medicine. After the study is completed, the de-identified, archived data will be transmitted to and stored at the NICHD Data and Specimen Hub (DASH), under the supervision of NIH/NICHD, for use by other researchers including those outside of the study. Permission to transmit data to NICHD DASH will be included in the informed consent.

There is no potential commercial uses or applications that may result from stored and shared data.

When the study is completed, access to study data will be provided through the NICHD DASH, through the use of written data sharing agreement with the study PIs and the requesting institution.

# Data Handling and Record Keeping

## Data Collection and Management Responsibilities

Data collection is the responsibility of the study staff at the site under the supervision of the PI. The PI is responsible for ensuring the accuracy, completeness, legibility, and timeliness of the data reported.

All source documents should be completed in a neat, legible manner to ensure accurate interpretation of data. Black ink is required to ensure clarity of reproduced copies. When making changes or corrections, cross out the original entry with a single line, and initial and date the change. DO NOT ERASE, OVERWRITE, OR USE CORRECTION FLUID OR TAPE ON THE ORIGINAL.

Copies of the electronic CRF (eCRF) will be provided for use as source documents and maintained for recording data for each participant enrolled in the study. Data reported in the eCRF derived from source documents should be consistent with the source documents or the discrepancies should be explained and captured in a progress note and maintained in the participant’s official electronic study record.

Research data will be entered into the data capture system provided by the NYULH Research Electronic Data Capture platform, called REDCap. REDCap is a HIPAA-compliant secure web application for building and managing online surveys and databases, specifically geared to support online and offline data capture for research studies and operations. The data system includes password protection and internal quality checks, such as automatic range checks, to identify data that appear inconsistent, incomplete, or inaccurate. Clinical data will be entered directly from the source documents.

### Data Collection Tools – Mobile Health Technology

**Products and Devices**

NYULH MCIT-managed iPhones and ipads . These devices will be used in accord with the Terms of Service (TOS) and/or the End User License Agreements (EULA) provided by the product or device vendor. Use of such products and devices may result in loss of privacy and risk of breach of confidentiality. These products and devices will only be used to collect study data with IRB approval and if the subject has agreed to all applicable Terms of Service and EULAs. The participant will be advised to read the full EULA or TOS before agreeing to use the product. Any risks associated are outlined in the informed consent.

Subjects will not need to download any apps or software to their personal devices.

## Study Records Retention

Study documents will be retained for the longer of 3 years after close out or 5 years after final reporting/publication. These documents should be retained for a longer period, however, if required by local regulations. No records will be destroyed without the written consent of the sponsor, if applicable. It is the responsibility of the sponsor to inform the investigator when these documents no longer need to be retained.

## Protocol Deviations

A protocol deviation is any noncompliance with the clinical trial protocol, GCP, or MOP requirements. The noncompliance may be either on the part of the participant, the investigator, or the study site staff. As a result of deviations, corrective actions are to be developed by the site and implemented promptly.

These practices are consistent with ICH E6:

- 4.5 Compliance with Protocol, sections 4.5.1, 4.5.2, and 4.5.3
- 5.1 Quality Assurance and Quality Control, section 5.1.1
- 5.20 Noncompliance, sections 5.20.1, and 5.20.2.

It is the responsibility of the site to use continuous vigilance to identify and report deviations within 10 working days of identification of the protocol deviation, or within 30 working days of the scheduled protocol-required activity. All deviations must be addressed in study source documents, reported to NIMHD Program Officials. Protocol deviations must be reported to the local IRB per their guidelines. The site PI/study staff is responsible for knowing and adhering to their IRB requirements. Further details about the handling of protocol deviations will be included in the MOP.

## Publication and Data Sharing Policy

This study will comply with the NIH Public Access Policy, which ensures that the public has access to the published results of NIH funded research. It requires scientists to submit final peer-reviewed journal manuscripts that arise from NIH funds to the digital archive PubMed Central upon acceptance for publication.

The International Committee of Medical Journal Editors (ICMJE) member journals have adopted a clinical trials registration policy as a condition for publication. The ICMJE defines a clinical trial as any research project that prospectively assigns human subjects to intervention or concurrent comparison or control groups to study the cause-and-effect relationship between a medical intervention and a health outcome. Medical interventions include drugs, surgical procedures, devices, behavioral treatments, process-of-care changes, and the like. Health outcomes include any biomedical or health-related measures obtained in patients or participants, including pharmacokinetic measures and adverse events. The ICMJE policy, and the Section 801 of the Food and Drug Administration Amendments Act of 2007, requires that all clinical trials be registered in a public trials registry such as ClinicalTrials.gov, which is sponsored by the National Library of Medicine. Other biomedical journals are considering adopting similar policies. For interventional clinical trials performed under NIH IC grants and cooperative agreements, it is the grantee’s responsibility to register the trial in an acceptable registry, so the research results may be considered for publication in ICMJE member journals. The ICMJE does not review specific studies to determine whether registration is necessary; instead, the committee recommends that researchers who have questions about the need to register err on the side of registration or consult the editorial office of the journal in which they wish to publish.

# Study Finances

## Funding Source

This study is financed through a grant from the US National Institutes of Health, National Institute for Minority Health and Health Disparities.

## Costs to the Participant

There are no costs to the participant.

## Participant Reimbursements or Payments

**Parent Participants**

Each participant dyad will be compensated for their time and travel with $25 for each survey and $35 for each of the two follow up in-person assessments for a total of up to $145 via gift cards. This incentive payment will be provided upon the completion of each study visit, and receipt of the payment is not contingent on successful completion of the study. These incentives are commonly used in research and considered a reasonable incentive without being coercive.

Parents who complete the qualitative interview will receive an additional $40.

**Care Provider Participants**

Care providers will receive a team lunch to thank them for their time completing all surveys. In addition, providers who complete the qualitative interview will receive an additional $40. This incentive payment will be provided upon the completion of each survey, and receipt of the payment is not contingent on successful completion of the study.

The study team will adhere to the [NYULH's Policy on Human Subject Payment](https://nyumc.ellucid.com/documents/view/6291/active/).

# Conflict of Interest Policy

The independence of this study from any actual or perceived influence, such as by the pharmaceutical industry, is critical. Therefore any actual conflict of interest of persons who have a role in the design, conduct, analysis, publication, or any aspect of this trial will be disclosed and managed. Furthermore, persons who have a perceived conflict of interest will be required to have such conflicts managed in a way that is appropriate to their participation in the trial. The study leadership in conjunction with the NIMHD has established policies and procedures for all study group members to disclose all conflicts of interest and will establish a mechanism for the management of all reported dualities of interest.

Any investigator who has a conflict of interest with this study (patent ownership, royalties, or financial gain greater than the minimum allowable by their institution, etc.) must have the conflict reviewed by the NYU Langone Conflict of Interest Committee with a Committee-sanctioned conflict management plan that has been reviewed and approved by the study sponsor prior to participation in this study. All NYULH investigators will follow the applicable conflict of interest policies.

# References

Reardon, S. F. (2011). The widening academic achievement gap between the rich and the poor: New evidence and possible explanations. In G. J. Duncan & R. J. Murnane (eds.), *Whither opportunity* (pp. 91-116). New York: Russell Sage Foundation.

1. Duncan, G. J., Ziol‐Guest, K. M., & Kalil, A. (2010). Early‐childhood poverty and adult attainment, behavior, and health. *Child Development, 81*(1), 306-325. https://doi.org/10.1111/j.1467-8624.2009.01396.x
2. Fernald, A., Marchman, V. A., & Weisleder, A. (2013). SES differences in language processing skill and vocabulary are evident at 18 months. *Developmental Science*, *16*(2), 234-248. PMCID: PMC3582035
3. Dreyer, B. P. (2020). Closing the gap: Interventions to ameliorate inequities in early brain development and school performance in poor children. *The Journal of Pediatrics*, *221*, 8-10. <https://doi.org/10.1016/j.jpeds.2020.02.004>
4. Shaw, D.S., Mendelsohn, A.L. & Morris, P.A. (2021). Reducing poverty-related disparities in child development and school readiness: The Smart Beginnings tiered prevention strategy that combines pediatric primary care with home visiting. *Clinical Child and Family Psychology Review,* 24, 669–683. PMCID: PMC8428206

Conger, R. D., Conger, K. J., & Martin, M. J. (2010). Socioeconomic status, family processes, and individual development. *Journal of Marriage and the Family*, *72*(3), 685–704. PMCID: PMC2910915

Linver, M. R., Brooks-Gunn, J., & Kohen, D. E. (2002). Family processes as pathways from income to young children's development. Developmental Psychology, 38(5), 719–734. https://doi.org/10.1037/0012-1649.38.5.719

Shonkoff, J. P., Garner, A. S.; Committee on Psychosocial Aspects of Child and Family Health; Committee on Early Childhood, Adoption, and Dependent Care; Section on Developmental and Behavioral Pediatrics. (2012). The lifelong effects of early childhood adversity and toxic stress. *Pediatrics, 129*(1), e232–e246.

Garner, A., Yogman, M., Committee on Psychosocial Aspects of Child and Family Health; Section on Developmental and Behavioral Pediatrics; Council on Early Childhood. Preventing childhood toxic stress: Partnering with families and communities to promote relational health. *Pediatrics, 148*(2):e2021052582

Love, J. M., Chazan-Cohen, R., Raikes, H., & Brooks-Gunn, J. (2013). What makes a difference: Early Head Start evaluation findings in a developmental context. *Monographs of the Society for Research in Child Development, 78*(1), 1-173. https://doi.org/10.1111/j.1540-5834.2012.00699.x

Olds, D. L., Kitzman, H., Cole, R., Robinson, J., Sidora, K., Luckey, D. W., ... & Holmberg, J. (2004). Effects of nurse home-visiting on maternal life course and child development: Age 6 follow-up results of a randomized trial. *Pediatrics*, *114*(6), 1550-1559. <https://doi.org/10.1542/peds.2004-0962>

Peacock-Chambers, E., Ivy, K., & Bair-Merritt, M. (2017). Primary care interventions for early childhood development: A systematic review. *Pediatrics, 140*(6), e20171661. https://doi.org/10.1542/peds.2017-1661

Johnston, B.D., Huebner, C.E., Tyll, L.T., Barlow, W.E., & Thompson, R.S. (2004). Expanding developmental and behavioral services for newborns in primary care: Effects on parental well-being, practice and satisfaction. *American Journal of Preventive Medicine, 26*(4), 356–366

Minkovitz, C. S., Hughart, N., Strobino, D., et al. (2003). A practice-based intervention to enhance quality of care in the first 3 years of life: The Healthy Steps for Young Children program. JAMA, *290*(23):3081–3091. https://doi.org/10.1001/jama.290.23.3081

Mendelsohn, A. L., Huberman, H. S., Berkule, S. B., Brockmeyer, C., Morrow, L. M., & Dreyer, B. P. (2011). Primary care strategies for promoting parent-child interactions and school readiness in at-risk families: The Bellevue Project for Early Language, Literacy, and Education Success. *Archives of Pediatrics & Adolescent Medicine,165*(1), 33-41. PMCID: PMC3095489

Cates, C. B., Weisleder, A., Johnson, S. B., Seery, A. M., Canfield, C. F., Huberman, H., ... & Mendelsohn, A. L. (2018). Enhancing parent talk, reading, and play in primary care: sustained impacts of the Video Interaction Project. *The Journal of Pediatrics*, *199*, 49-56. PMCID: PMC6063788

1. Mendelsohn, A. L., Cates, C. B., Weisleder, A., et al. (2018). Reading aloud, play, and social-emotional development. *Pediatrics*, *141*(5). PMCID: PMC5914489

Glasgow, R. E., Vogt, T. M., & Boles, S. M. (1999). Evaluating the public health impact of health promotion interventions: The RE-AIM framework. *American Journal of Public Health, 89*,1322-7. PMCID: PMC1508772

Damschroder, L. J., Aron, D. C., Keith, R. E., Kirsh, S. R., Alexander, J. A., & Lowery, J. C. (2009). Fostering implementation of health services research findings into practice: a consolidated framework for advancing implementation science. *Implementation Science*, *4*(1), 1-15. PMCID: PMC2736161

1. Gross, R. S., Messito, M. J., Klass, P., Canfield, C. F., Yin, H. S., Morris, P. A., Shaw, D. S., Dreyer, B. P., & Mendelsohn, A. L. (2021). Integrating health care strategies to prevent poverty-related disparities in development and growth: Addressing core outcomes of early childhood. *Academic Pediatrics, 21*(8S):S161-S168. PMCID: PMC8574213

Duncan, G. J., Morris, P. A., & Rodrigues, C. (2011). Does money really matter? Estimating impacts of family income on young children's achievement with data from random-assignment experiments. *Developmental Psychology, 47*(5):1263-79. PMCID: PMC3208322

Campbell, F., Conti, G., Heckman, J. J., Moon, S. H., Pinto, R., Pungello, E., & Pan, Y. (2014). Early childhood investments substantially boost adult health. *Science*, *343*(6178), 1478-1485. PMCID: PMC4028126

Evans, G. W. (2016). Childhood poverty and adult psychological well-being. *Proceedings of the National Academy of Sciences*, *113*(52), 14949-14952. PMCID: PMC5206566

United Nations. (2015). Transforming our world: The 2030 agenda for sustainable development. Retrieved from https://sdgs.un.org/publications/transforming-our-world-2030-agenda-sustainable-development-17981

Gennetian, L., Castells, N., & Morris, P. A. (2010). Meeting the basic needs of children: Does income matter? *Children and Youth Services Review, 32*(9), 1138-1148. PMCID: PMC2913899

Dahl, G., & Lochner, L. (2008). The impact of family income on child achievement: Evidence from the Earned Income Tax Credit (NBER Working Paper No. 14599). Cambridge, MA: National Bureau of Economic Research.

Milligan, K., & Stabile, M. (2008). Do child tax benefits affect the well-being of children? Evidence from Canadian child benefit expansions (NBER Working Paper No. 14264). Cambridge, MA: National Bureau of Economic Research.

Duncan, G., & Brooks-Gunn, J. (1997). The effects of poverty on children. *The Future of Children, 7*(2), 55-71.

Duncan, G., Brooks-Gunn, J., Yeung, J., et al. (1998). How much does childhood poverty affect the life chances of children? *American Sociological Review, 63*, 406-23.

Halle, T., Forry, N., Hair, E., Perper, K., Wandner, L., Wessel, J., et al. (2009). *Disparities in early learning and development: Lessons from the Early Childhood Longitudinal Study–Birth Cohort (ECLS-B).* Washington, DC: Child Trends.

Koball, H., Moore, A, & Hernandez, J. (2021). Basic facts about low-income children: Children under 9 years, 2019. New York: National Center for Children in Poverty, Bank Street College of Education.

Isaacs, J. B. (2012). Starting school at a disadvantage: The school readiness of poor children. Washington, DC: Brookings Institution

Duncan, G. J., Dowsett, C. J., Claessens, A., Magnuson, K., Huston, A. C., Klebanov, P., ... & Sexton, H. (2007). School readiness and later achievement. *Developmental Psychology, 43*(6), 1428. https://doi.org/10.1037/0012-1649.43.6.1428

Reardon, S. F., & Portilla, X. A. (2016). Recent trends in income, racial, and ethnic school readiness gaps at kindergarten entry. *AERA Open, 2*(3), 1-18. https://doi.org/10.1177/2332858416657343

Comeau, J., & Boyle, M. H. (2017). Patterns of poverty exposure and children's trajectories of externalizing and internalizing behaviors. *SSM - Population Health*, *4*, 86–94. PMCID: PMC5769125

CDC, National Center for Injury Prevention and Control, Division of Violence Prevention, April 6, 2021. https://www.cdc.gov/violenceprevention/aces/fastfact.html

Merrick, M. T., Ford, D. C., Ports, K. A., & Guinn, A. S. (2018). Prevalence of adverse childhood experiences from the 2011-2014 Behavioral Risk Factor Surveillance System in 23 States. *JAMA Pediatrics, 172*(11), 1038-1044. PMCID: PMC6248156

Child Trends. (2019). Adverse experiences. Retrieved from https://www.childtrends.org/?indicators=adverse-experiences

Shonkoff, J. P., & Phillips, D. A. (eds). (2000). From neurons to neighborhoods: The science of early childhood development. National Research Council and Institute of Medicine. Washington DC: National Academy Press.

Peterson, C., Florence, C., & Klevens, J. (2018). The economic burden of child maltreatment in the United States, 2015. *Child Abuse and Neglect, 86*, 178-183. PMCID: PMC6289633

Peterson, C., DeGue, S., Florence, C., & Lokey, C. (2017). Lifetime economic burden of rape in the United States. *American Journal of Preventive Medicine, 52*(6), 691-701. PMCID: PMC5438753

Peterson, C., Kearns, M. C., McIntosh, W. L., Estefan, L. F., Nicolaidis, C., McCollester, K. E., Gordon, A., & Florence, C. (2018). Lifetime economic burden of intimate partner violence among U.S. adults. *American Journal of Preventive Medicine, 5*5(4), 433-444. PMCID: PMC6161830

Centers for Disease Control and Prevention. (2019). Cost of Injury. Web-based Injury Statistics Query and Reporting System (WISQARS). Atlanta, GA: Centers for Disease Control and Prevention, National Center for Injury Prevention and Control. Retrieved from http://www.cdc.gov/injury/wisqars/

Leeb, R. T., Lewis, T., & Zolotor, A. J. (2011). A review of the physical and mental health consequences of child abuse and neglect and implications for practice. *American Journal of Lifestyle Medicine, 5*(5), 454-468

Hillis, S. D., Anda, R. F., Dube, S. R., Felitti, V. J., Marchbanks, P. A., & Marks, J. S. (2004). The association between adverse childhood experiences and adolescent pregnancy, long-term psychosocial outcomes, and fetal death. *Pediatrics, 113*(2), 320-327.

Metzler, M., Merrick, M. T., Klevens, J., Ports, K. A., & Ford, D. C. (2017). Adverse childhood experiences and life opportunities: Shifting the narrative. *Children and Youth Services Review, 72*, 141-149.

Edwards, V. J., Anda, R. F., Dube, S. R., Dong, M., Chapman, D. F., & Felitti, V. J. (2005). The wide-ranging health consequences of adverse childhood experiences. In Kathleen Kendall-Tackett and Sarah Giacomoni (eds.) *Victimization of Children and Youth: Patterns of Abuse, Response Strategies*. Kingston, NJ: Civic Research Institute.

Gilbert, L. K., Breiding, M. J., Merrick, M. T, Parks, S. E, Thompson, W. W., Dhingra, S. S., & Ford, D. C. (2015). Childhood adversity and adult chronic disease: An update from ten states and the District of Columbia, 2010. *American Journal of Preventive Medicine, 48*(3), 345-349.

Chapman, D. P., Anda, R. F., Felitti, V. J., Dube, S. R., Edwards, V. J., Whitfield, C. L. (2004). Adverse childhood experiences and the risk of depressive disorders in adulthood. *Journal of Affective Disorders, 82*, 217-225.

Sege, R. D., & Harper Browne, C. (2017). Responding to ACEs with HOPE: Health Outcomes From Positive Experiences. *Academic Pediatrics, 17*(7S):S79–S85

Bethell, C., Jones, J., Gombojav, N., Linkenbach, J., & Sege, R. (2019). Positive childhood experiences and adult mental and relational health in a statewide sample: Associations across adverse childhood experiences levels. *JAMA Pediatrics, 173*(11):e193007. PMCID: PMC6735495

National Scientific Council on the Developing Child. (2015). Supportive relationships and active skill-building strengthen the foundations of resilience: Working Paper No. 13. Cambridge, MA: National Scientific Council on the Developing Child. Retrieved from https://developingchild.harvard.edu/resources/supportive-relationships-and-active-skill-building-strengthen-the-foundations-of-resilience/.

Bethell, C. D., Gombojav, N., & Whitaker, R. C. (2019). Family resilience and connection promote flourishing among US children, even amid adversity. *Health Affairs*, *38*(5), 729-737.

Yamaoka, Y., & Bard, D. E. (2019). Positive parenting matters in the face of early adversity. *American Journal of Preventive Medicine*, *56*(4), 530-539.

Willis, D., Chavez, S., Lee, J., Hampton, P., & Fine, P (2020). Early relational health national survey: What we’re learning from the field. Washington, DC: Center for the Study of Social Policy. Retrieved from https://cssp.org/resource/early-relational-health-survey.

Brody, G. H., Yu, T., & Beach, S. R. (2016). Resilience to adversity and the early origins of disease. *Developmental Psychopathology, 28*(4, pt 2), 1347–1365. PMCID: PMC5079770

Flouri, E., Midouhas, E., Joshi, H., & Tzavidis, N. (2015). Emotional and behavioural resilience to multiple risk exposure in early life: The role of parenting. *European Child & Adolescent Psychiatry, 24*(7):745–755

Crouch, E., Radcliff, E., Brown, M., & Hung, P. (2019). Exploring the association between parenting stress and a child's exposure to adverse childhood experiences (ACEs). *Children and Youth Services Review, 102*, 186-192. PMCID: PMC7266302

McLoyd, V. C. (2011). How money matters for children’s socioemotional adjustment: Family processes and parental investment. In *Health disparities in youth and families* (pp. 33-72). Springer, New York, NY.

Sanders, M.R., Ralph, A., Sofronoff, K., Gardiner, P., Thompson, R., Dwyer, S., & Bidwell, K. (2008). Every Family: A population approach to reducing behavioral and emotional problems in children making the transition to school. *Journal of Primary Prevention, 29*, 197-222.

HRSA, Maternal and Child Health Bureau (2017). The maternal, infant, and early childhood home visiting program: Partnering with parents to help children succeed. Retrieved from https://mchb.hrsa.gov/sites/default/files/mchb/MaternalChildHealthInitiatives/HomeVisiting/pdf/programbrief.pdf

1. Muñiz, E. I., Silver, E. J., Stein, R. E. K. (2014). Family routines and social-emotional school readiness among preschool-age children. *Journal of Developmental & Behavioral Pediatrics, 35*(2), 93-99. https://doi.org/10.1097/DBP.0000000000000021
2. Cabrera, N. J., Karberg, E., Malin, J. L., & Aldoney, D. (2017). The magic of play: Low‐income mothers’ and fathers’ playfulness and children’s emotion regulation and vocabulary skills. *Infant Mental Health Journal*, *38*(6), 757-771.
3. Vesely, C. K., Brown, E. L., & Mahatmya, D. (2013). It takes two: Sensitive caregiving across contexts and children's social, emotional, and academic outcomes. *Early Education & Development*, *24*(7), 960-978.

Raikes, H., Alexander Pan, B., Luze, G., Tamis‐LeMonda, C. S., Brooks‐Gunn, J., Constantine, J., ... & Rodriguez, E. T. (2006). Mother–child bookreading in low‐income families: Correlates and outcomes during the first three years of life. *Child Development, 77*(4), 924-953. https://doi.org/10.1111/j.1467-8624.2006.00911.x

Golinkoff, R. M., Hoff, E., Rowe, M. L., Tamis‐LeMonda, C. S., & Hirsh‐Pasek, K. (2019). Language matters: Denying the existence of the 30‐million‐word gap has serious consequences. *Child Development, 90*(3), 985-992. https://doi.org/10.1111/cdev.13128

Mistry, R. S., Benner, A. D., Biesanz, J. C., Clark, S. L., & Howes, C. (2010). Family and social risk, and parental investments during the early childhood years as predictors of low-income children's school readiness outcomes. *Early Childhood Research Quarterly, 25*(4), 432-449. https://doi.org/10.1016/j.ecresq.2010.01.002

Gennetian, L. A., Duncan, G., Fox, N. A., Magnuson, K., Halpern-Meekin, S., Noble, K. G., & Yoshikawa, H. (2022). *Unconditional Cash and Family Investments in Infants: Evidence from a Large-Scale Cash Transfer Experiment in the US* (No. w30379). National Bureau of Economic Research.

National Research Council and Institute of Medicine Committee on the Prevention of Mental Disorders and Substance Abuse Among Children, Youth, and Young Adults: Research Advances and Promising Interventions. (2009). *Preventing mental, emotional, and behavioral disorders among young people: Progress and possibilities*. National Academies Press.

Mendelsohn, A. L., Valdez, P. T., Flynn, V., Foley, G. M., Berkule, S. B., Tomopoulos, S., ... & Dreyer, B. P. (2007). Use of videotaped interactions during pediatric well-child care: impact at 33 months on parenting and on child development. *Journal of Developmental & Behavioral Pediatrics*, 28(3), 206-212. PMCID: PMC3083927

Piotrowski, C. C., Talavera, G. A., & Mayer, J. A. (2009). Healthy steps: a systematic review of a preventive practice-based model of pediatric care. *Journal of Developmental & Behavioral Pediatrics*, 30(1), 91-103.

Shaw, D. S., Connell, A., Dishion, T. J., Wilson, M. N., & Gardner, F. (2009). Improvements in maternal depression as a mediator of intervention effects on early childhood problem behavior. *Development and psychopathology*, *21*(2), 417-439. PMCID: PMC2770003

Shonkoff JP. Rethinking the definition of evidence-based interventions to promote early childhood development. *Pediatrics*; 2017:140(6).

Child Trends Databank. (2018). Well-child visits. https://www.childtrends.org/indicators/well-child-visits

Bouchery, E. (2012). Utilization of well-child care among Medicaid-enrolled children. Mathematica Policy Research, Centers for Medicare & Medicaid Services (Brief 10).

Moseley, K. L., Freed, G. L., & Goold, S. D. (2011). Which sources of child health advice do parents follow?. *Clinical Pediatrics*, *50*(1), 50-56

Center for the Study of Social Policy. (2022). Policy Change to Promote Early Relational Health. https://cssp.org/resource/policy-change-to-promote-erh/

InNK Marks National Advisory Team. (2020). Framework for Transforming Children’s Health Care. https://www.inckmarks.org/docs/guidingframework/NACSTATEMENTTRANSFORMATIONpdf.pdf

Gross, D., Julion, W., & Fogg, L. (2004). What motivates participation and dropout among low-income urban families of color in a prevention intervention? Family Relations, 50, 246–254. https://doi.org/10. 1111/j.1741-3729.2001.00246.x.

Ingoldsby, E. M. (2010). Review of interventions to improve family engagement and retention in parent and child mental health programs. Journal of Child and Family Studies, 19, 629–645. PMCID: PMC2930770

Baker, C. N., Arnold, D. H., & Meagher, S. (2011). Enrollment and attendance in a parent training prevention program for conduct problems. Prevention Science, 12, 126–138. https://doi.org/10.1007/ s11121-010-0187-0.

Reyno, S. M., & McGrath, P. J. (2006). Predictors of parent training efficacy for child externalizing behavior problems—A metaanalytic review. Journal of Child Psychology and Psychiatry, 47, 99–111. https://doi.org/10.1111/j.1469-7610.2005.01544.x.

Ader, J., Stille, C. J., Keller, D., Miller, B. F., Barr, M. S., & Perrin, J. M. (2015). The medical home and integrated behavioral health: Advancing the policy agenda. *Pediatrics, 135*(5), 909-917.

Blount, A. (2003). Integrated primary care: Organizing the evidence. *Families Systems and Health, 21*, 121-134.

Hacker, K. A., Penfold, R. B., Arsenault, L. N., Zhang, F., Soumerai, S. B., & Wissow, L. S. (2015). Effect of pediatric behavioral health screening and co-located services on ambulatory and inpatient utilization. *Psychiatric Services, 66*, 1141–1148. PMCID: PMC4633707

1. Campo, J. V., Shafer, S., Strohm, J., Lucas, A., Cassesse, C. G., Shaeffer, D., & Altman, H. (2005). Pediatric behavioral health in primary care: A collaborative approach. *Journal of the American Psychiatric Nurses Association*, *11*(5), 276-282.
2. Ward-Zimmerman, B., & Cannata, E. (2012). Partnering with pediatric primary care: lessons learned through collaborative colocation. *Professional Psychology: Research and Practice*, *43*(6), 596.

Powell, D. S., & Batsche, C. J. (1997). A strength-based approach in support of multi-risk families: Principles and issues. *Topics in Early Childhood Special Education,17*(1):1-26. https://doi.org/10.1177/027112149701700105

Heinicke, C. M., Fineman, N. R., Ruth, G., Recchia, S. L., Guthrie, D., & Rodning, C. (1999). Relationship-based intervention with at-risk mothers: Outcome in the first year of life. *Infant Mental Health Journal, 20*(4):349-374. https://doi.org/10.1002/(SICI)1097-0355(199924)20:4<349::AID-IMHJ1>3.0.CO;2-X

Dishion, T. J., & Stormshak, E. A. (2007). Intervening in Children’s Lives: An Ecological, Family-Centered Approach to Mental Health Care. Washington: American Psychological Association. https://doi.org/10.1037/11485-000

Dawson-McClure, S., Calzada, E. J., & Brotman, L. M. (2017). Engaging parents in preventive interventions for young children: Working with cultural diversity within low-income, urban neighborhoods. *Prevention Science, 18*(6):660-670. doi:10.1007/s11121-017-0763-7

Oral, R., Ramirez, M., Coohey, C., et al. (2016). Adverse childhood experiences and trauma informed care: The future of health care. *Pediatric Research, 79*(1-2):227-233. doi:10.1038/pr.2015.197

1. Valado, T., Tracey, J., Goldfinger, J., & Briggs, R. (2019). HealthySteps: Transforming the promise of pediatric care. *The Future of Children*, *29*(1), 99-122.

Zero to Three Foundation. (n.d.). *Zero to Three*. https://www.zerotothree.org/

1. Mendelsohn, A. L., Cates, C. B., Weisleder, A., Berkule, S. B., & Dreyer, B. P. (2013). Promotion of early school readiness using pediatric primary care as an innovative platform. *Zero to Three*, *34*(1), 29-40.
2. Kumar, M., Huang, K. Y., Othieno, C., Wamalwa, D., Hoagwood, K., Unutzer, J., . . . McKay, M. (2020). Implementing combined WHO mhGAP and adapted group interpersonal psychotherapy to address depression and mental health needs of pregnant adolescents in Kenyan primary health care settings (INSPIRE): a study protocol for pilot feasibility trial of the integrated intervention in LMIC settings. *Pilot Feasibility Stud, 6*, 136. PMCID: PMC7507720
3. Kumar, M., Verdeli, H., Saxena, S., Petersen, I., Huang, K. Y., Othieno, C., . . . McKay, M. (2022). Modifying Group Interpersonal Psychotherapy for Peripartum Adolescents in Sub-Saharan African Context: Reviewing Differential Contextual and Implementation Considerations. *Clinical Medicine Insights: Psychiatry, 13*. doi:10.1177/1179557322107557.
4. Huang, K. Y., Nakigudde, J., Kisakye, E. N., Sentongo, H., Dennis-Tiwary, T. A., Tozan, Y., . . . Brotman, L. M. (2022). Advancing scalability and impacts of a teacher training program for promoting child mental health in Ugandan primary schools: protocol for a hybrid-type II effectiveness-implementation cluster randomized trial. *Int J Ment Health Syst, 16*(1), 28. PMCID: PMC9206883
5. Huang, K.-Y., Nakigudde, J., & Brotman, L. (2015). Use of task-shifting to scale-up child mental health services in low-resource Ugandan schools: Role of contextual factors on program implementation. *Implementation Science, 10 (Suppl 1)*. doi:10.1186/1748-5908-10-S1-A23
6. Huang, K. Y., Kwon, S. C., Cheng, S., Kamboukos, D., Shelley, D., Brotman, L. M., . . . Hoagwood, K. (2018). Unpacking Partnership, Engagement, and Collaboration Research to Inform Implementation Strategies Development: Theoretical Frameworks and Emerging Methodologies. *Front Public Health, 6*, 190. PMCID: PMC6050404
7. Huang, K.Y. ., Cheng, S., Yee, S., Hoagwood, K., McKay, M., Shelley, D., Ogedegbe, G., Brotman, L.M. (Dec 2016) Integrating Evidence-Based Pediatric Behavioral Health Services into Primary and Community Settings: Pragmatic Strategies and Lessons Learned from Literature Review and Global Implementation Projects. Paper presented at the Global Dissemination and Implementation Symposium at NIH 9^th^ Annual Conference on the Science of Dissemination and Implementation 2016 Meeting, Washington DC, MD.
8. Huang, K.Y. & Yee, S., Cheng, S., Hoagwood, K. (Dec 2015) Integrating evidence-based child behavioral and developmental preventive interventions into community primary care settings: current practices and predictors for utilization. Paper presented as a poster at the 8^th^ Annual Conference on the Science of Dissemination and Implementation 2015 Meeting, Washington DC, MD.
9. Yee, S. (2017). Integrating behavioral and developmental evidence based interventions & practices in diverse primary care settings: Barriers and strategies. School of Public Health, SUNY Downstate Medical Center. Dissertation.
10. Perez Jolles, M., Lengnick-Hall, R., & Mittman, B. S. (2019). Core functions and forms of complex health interventions: A patient-centered medical home illustration. *Journal of General Internal Medicine, 34*(6), 1032-8. PMCID: PMC6544719
11. Caughy, M. O. B., Huang, K. Y., Miller, T., & Genevro, J. L. (2004). The effects of the Healthy Steps for Young Children Program: Results from observations of parenting and child development. *Early Childhood Research Quarterly*, *19*(4), 611-630.
12. Minkovitz, C. S., Strobino, D., Mistry, K. B., Scharfstein, D. O., Grason, H., Hou, W., ... & Guyer, B. (2007). Healthy Steps for Young Children: Sustained results at 5.5 years. *Pediatrics*, *120*(3), e658-e668.

Minkovitz, C., Strobino, D., Hughart, N., Scharfstein, D., Guyer, B., and the Healthy Steps Evaluation Team. (2001). Early Effects of the Healthy Steps for Young Children Program. *Archives of Pediatrics & Adolescent Medicine*,*155*(4):470–479. https://doi.org/10.1001/archpedi.155.4.470

Guyer, B., Barth, M., Bishai, D., Caughy, M., Clark, B., Burkom, D., Genevro, J., Grason, H., Hou, W., Huang, K-Y., Hughart, N., Snow Jones, A., McLearn, K.T., Miller, T., Minkovitz, C., Scharfstein, D., Stacy, H., Strobino, D., Szanton, E., & Tang, C. (2003). Healthy Steps: The first three years: The Healthy Steps for Young Children Program National Evaluation. Retrieved from https://ztt-healthysteps.s3.amazonaws.com/ documents/139/attachments/2003_ H_National_Evaluation_Report.pdf?1539967

1. Briggs, R. D., Silver, E.J., Krug, L.M., Mason, Z.S., Schrag, R.D.A., Chinitz, S., & Racine, A D. (2014). Healthy Steps as a moderator: The impact of maternal trauma on child social-emotional development. *Clinical Practice in Pediatric Psychology, 2*(2), 166–175

Till, L. et al. (2017). HealthySteps Implementation and Outcome Study Evaluation Report Washington, DC: Zero to Three.

1. Mendelsohn, A. L., Dreyer, B. P., Flynn, V., Tomopoulos, S., Rovira, I., Tineo, W., ... & Nixon, A. F. (2005). Use of videotaped interactions during pediatric well-child care to promote child development: A randomized, controlled trial. *Journal of Developmental and Behavioral Pediatrics, 26*(1), 34. PMCID: PMC4435697

Canfield, C. F., Weisleder, A., Cates, C. B., et al. (2015). Primary care parenting intervention and its effects on the use of physical punishment among low-income parents of toddlers. *Journal of Developmental & Behavioral Pediatrics*, *36*(8):586-593. PMCID: PMC4586371

1. Mendelsohn, A. L., Dreyer, B. P., Brockmeyer, C., Berkule-Silberman, S. B., Huberman, H. S., Tomopoulos, S. (2011). Randomized controlled trial of primary care pediatric parenting programs: Effect on reduced media exposure in infants, mediated through enhanced parent-child interaction. *Archives of Pediatrics & Adolescent Medicine*,*165*(1), 42-48. PMCID: PMC3083922
2. Burwick, A., & Zaveri, H. (2014). Costs of early childhood home visiting: An analysis of programs implemented in the supporting evidence-based home visiting to prevent child maltreatment initiative. Mathematica Policy Research. https://econpapers.repec.org/paper/mprmprres/9474fe2a20a24941810fce8a203cfc58.htm
3. Miller, E. B., Canfield, C. F., Morris, P. A., Shaw, D. S., Cates, C. B., & Mendelsohn, A. L. (2020). Sociodemographic and psychosocial predictors of VIP attendance in smart beginnings through 6 months: Effectively targeting at-risk mothers in early visits. *Prevention Science*, *21*(1), 120-130. PMCID: PMC6960338
4. Canfield, C. F., Miller, E. B., Zhang, Y., Shaw, D. S., Morris, P. A., & Mendelsohn, A. L. Tiered universal and targeted early childhood preventive interventions: Enhancing attendance across families with varying needs and strengths. Manuscript under review.
5. Mendelsohn, A., Matalon, M., & Canfield, C. *VIP Parent Satisfaction Surveys*. [Unpublished data]. NYU Grossman School of Medicine, Department of Pediatrics.

Hughes, S., Herrera-Mata, L., & Dunn, J. (2014). Impact of Healthy Steps on developmental referral rates. *Family Medicine, 46*(10), 788-79

Johnston, B.D., Huebner, C.E., Anderson, M.L., Tyll, L.T., & Thompson, R.S. (2006). Healthy Steps in an integrated delivery system: Child and parent outcomes at 30 months. *Archives of Pediatrics & Adolescent Medicine, 160*(8), 793–800.

1. Gross, R. S., Briggs, R. D., Hershberg, R.S., Silver, E.J., Velazco, N.K., Hauser, N.R., & Racine, A.D. (2015). Early child social-emotional problems and child obesity: Exploring the protective role of a primary care-based general parenting intervention. *Journal of Developmental & Behavioral Pediatrics, 36*(8), 594–604.
2. Katzow, M., Canfield, C., Gross, R., et al. (2019). Maternal depressive symptoms and perceived picky eating in a low-income, primarily Hispanic sample. *Journal of Developmental & Behavioral Pediatrics*, *40*(9), 706-715. PMCID: PMC6878153
3. Miller, E. B., Whipps, M. D. M., Bogen, D. L., Morris, P. A., Mendelsohn, A. L., Shaw, D. S, & Gross, R. S. (2022). Collateral benefits from a school-readiness intervention on breastfeeding: A cross-domain impact evaluation. *Maternal & Child Nutrition*. PMC Journal – In Process

Berkule, S. B., Cates, C. B., Dreyer, B. P., Huberman, H. S., Arevalo, J., Burtchen, N., Weisleder, A., & Mendelsohn, A. L. (2014). Reducing maternal depressive symptoms through promotion of parenting in pediatric primary care. *Clinical Pediatrics 53*(5), 460-9. PMCID: PMC4435690

Cates, C. B., Weisleder, A., Dreyer, B. P., et al. (2016). Leveraging healthcare to promote responsive parenting: Impacts of the Video Interaction Project on parenting stress. *Journal of Child & Family Studies, 25*(3):827-835. PMCID: PMC4847426

Gross, R. S., Canfield, C. F., Hails, K., Whipps. M., Miller, E. B., Bogen, D. L., Morris, P., Shaw, D., & Mendelsohn, A. L. (202). Early cognitive home environment and obesity-promoting feeding styles at 24 months in low-income families: General parenting intervention impacts. Platform Presentation at Pediatr Acad Soc Meet Philadelphia, PA.

1. Roby, E., Miller, E. B., Shaw, D. S., Morris, P., Gill, A., Bogen, D. L., ... & Mendelsohn, A. L. (2021). Improving parent-child interactions in pediatric health care: A two-site randomized controlled trial. *Pediatrics*, *147*(3). PMCID: PMC7924140

Miller, E. B., Roby, E., Zhang, Y., Coskun, L., Rosas, J. M., Scott, M. A., Shaw, D.S., Mendelsohn, A. L., & Morris, P. A. Promoting cognitive stimulation in low-income parents across infancy and toddlerhood: A randomized clinical trial. Manuscript submitted for publication.

1. Mendelsohn, A. L., Cates, C. B., Huberman, H. S., Johnson, S. B., Govind, P., Kincler, N., ... & Dreyer, B. P. (2020). Assessing the impacts of pediatric primary care parenting interventions on EI referrals through linkage with a public health database. *Journal of Early Intervention*, *42*(1), 69-82.
2. Weisleder, A., Cates, C. B., Dreyer, B. P., et al. (2016). Promotion of positive parenting and prevention of socioemotional disparities. *Pediatrics,137*(2). PMCID: PMC4732361
3. Academic Pediatric Society, Continuity Research Network (CORNET). Retrieved from https://www.academicpeds.org/groups-networks/research-networks/cornet/
4. HealthySteps Annual Report. (2020). Retrieved from https://www.healthysteps.org/news-resources/annual-report/

Herman-Smith, R. L. (2013). Early childhood interventionists' perceptions of the Child Abuse Prevention and Treatment Act: Provider characteristics and organizational climate. Early Education & Development, 24(3), 393-407.

Waltz, T. J., Powell, B. J., Matthieu, M. M., Damschroder, L. J., Chinman, M. J., Smith, J. L., ... & Kirchner, J. E. (2015). Use of concept mapping to characterize relationships among implementation strategies and assess their feasibility and importance: results from the Expert Recommendations for Implementing Change (ERIC) study. *Implementation Science*, *10*(1), 1-8. PMCID: PMC4527340

Glaser, B. G. (1965). The constant comparative method of qualitative analysis. *Social Problems, 12*(4), 436-445.

Flick, U. (1992) Triangulation revisited – strategy of or alternative to validation of qualitative data, *Journal for the Theory of Social Behavior, 22*, 175–197.

1. Connor, K. I., Siebens, H. C., Mittman, B. S., Ganz, D. A., Barry, F., McNeese-Smith, D. K., et al. (2022). Implementation fidelity of a nurse-led RCT-tested complex intervention, care coordination for health promotion and activities in Parkinson's disease (CHAPS) in meeting challenges in care management. *BMC Neurology, 22*(1):36. PMCID: PMC8785022
2. Tomopoulos, S. (2021). *Partnerships for Early Childhood Development, Year 3 Final Grant Report*. United Hospital Fund.
3. NICHD Early Child Care Research Network. (1999). Child care and mother-child interaction in the first three years of life. *Developmental Psychology, 35*(6), 1399-413.
4. Tamis-LeMonda, C., Rodriguez, V., Shannon, J., Hannibal, B., Ahuja, P., & Spellmann, M. Caregiver-Child Affect, Responsiveness, and Engagement Scale (C-CARES) Fourteen-Month Version, New York University. Retrieved from http://steinhardt.nyu.edu/crcde/pdf/NYU_CCARES14mo.pdf
5. Clark, R. (1999). The parent-child Early Relational Assessment: A factorial validity study. *Educational and Psychological Measurement, 59*(5), 821-46.
6. Shaw, D. S., Winslow, E. B., Owens, E. B., Vondra, J. I., Cohn, J. F., & Bell, R. Q. (1998). The development of early externalizing problems among children from low-income families: A transformational perspective. *Journal of Abnormal Child Psychology, 26*, 95-107.
7. Weaver, C., Shaw, D. S., Crossan, J., Dishion, T. J., & Wilson, M. N. (2014). Parent-child conflict and early childhood adjustment in two-parent low-income families: Parallel developmental processes. *Child Psychiatry & Human Development, 46*, 94-107. PMCID: PMC4523123
8. Dreyer, B. P., Mendelsohn, A. L., Tamis‐LeMonda, C. S. (1996). Assessing the child’s cognitive home environment through parental report: Reliability and validity. *Infant and Child Development, 5*(4), 271-87
9. Cox, J. L., Chapman, G., Declan, M., Jones, P. (1995). Validation of the Edinburgh Postnatal Depression Scale (EPDS) in non-postnatal women. *Journal of Affective Disorders, 39*(3), 185-189. https://doi.org/10.1016/0165-0327(96)00008-0.
10. Nielsen, M. G., Ørnbøl, E., Vestergaard, M., Bech, P., Larsen, F. B., Lasgaard, M., & Christensen, K. S. (2016). The construct validity of the Perceived Stress Scale. *Journal of psychosomatic research*, *84*, 22-30.
11. Dumka, L., Stoerzinger, H., Jackson, K., & Roosa, M. (1996). Examination of the cross-cultural and cross-language equivalence of the Parenting Self-Agency Measure. *Family Relations, 45*, 216-222.
12. Crnic, K. A., Greenberg, M. T., Rogozin, A. S., Robinson, N. M., & Basham, R. B. (1983). Effects of stress and social support on mothers and premature and full-term infants. *Child Development, 54*(1), 209-17. https://doi.org/10.2307/1129878.
13. Spanier, G. B. (1976). Measuring dyadic adjustment: New scales for assessing the quality of marriage and similar dyads. *Journal of Marriage and the Family*, 15-28.
14. McEachern, A., Dishion, T. J., Shaw, D. S., Wilson, M. N., & Gardner, F. (2012). Parenting young children (PARYC): Validation of a self-report parenting measure. *Journal of Child & Family Studies, 21*, 498-511. PMCID: PMC3412343
15. Socolar, R., Savage, E., Devellis, R. F., et al. (2004). The discipline survey: A new measure of parental discipline. *Ambulatory Pediatrics, 4*(2),166-173.
16. Gartstein, M., & Rothbart, M. K. Studying infant temperament via the revised Infant Behavior Questionnaire. *Infant Behavior & Development, 26*(1), 64-86.
17. Briggs-Gowan, M. J., Carter, A. S., Irwin, J. R., Wachtel, K., & Cicchetti, D. V. (2004). The Brief Infant-Toddler Social and Emotional Assessment: Screening for social-emotional problems and delays in competence. *Journal Pediatric Psychology, 29*(2), 143-55.
18. Achenbach, T. M., & Rescorla, L. A. (2000). Manual for the ASEBA preschool forms & profiles. Burlington, VT: University of Vermont, Research Center for Children, Youth & Families.
19. Mundy, P., Delgado, C., Block, J., Venezia, M., Hogan, A., & Seibert, J. (2003). *A manual for the abridged Early Social Communication Scales*. Coral Gables, FL: University of Miami.
20. Fenson, L. (2007). MacArthur-Bates Communicative Development Inventories. Baltimore, MD: Paul H. Brookes Publishing Company.
21. Gardner, M. F. (1990a). Expressive One-Word Picture Vocabulary Test-Revised. Novato, CA: Academic Therapy Publications.
22. Gardner, M. F. (1990b). Receptive One-Word Picture Vocabulary Test-Revised. Novato, CA: Academic Therapy Publications.

Weiner, B.J., Lewis, C.C., Stanick, C. *et al.* (2017). Psychometric assessment of three newly developed implementation outcome measures. *Implementation Science,* 12(108). PMCID: PMC5576104

1. Aarons, G. A., Glisson, C., Hoagwood, K., Kelleher, K., Landsverk, J., & Cafri, G. (2010). Psychometric properties and U.S. National norms of the Evidence-Based Practice Attitude Scale (EBPAS). *Psychological Assessment, 22*(2), 356–365. PMCID: PMC3841109

Little, R. J. A., & Rubin, D. B. (2002). *Statistical Analysis with Missing Data*. Wiley.

1. White, I. R., Royston, P., & Wood, A. M. (2011). Multiple imputation using chained equations: Issues and guidance for practice. *Statistics in Medicine*, *30*(4):377-399. https://doi.org/10.1002/sim.4067
2. Jurs, S., & Glass, G. (1971). The effect of experimental mortality on the internal and external validity of the randomized comparative experiment. *Journal of Experimental Education, 40*(1):62-66.
3. Hedeker, D., & Gibbons, R. D. (2006). *Longitudinal data analysis (Vol. 451)*. John Wiley & Sons.

MacKinnon, D. P., Fairchild, A. J., & Fritz, M. S. (2007). Mediation analysis. *Annual Review of Psychology, 58*, 593-614. PMCID: PMC2819368

Hayes, A. F. (2009). Beyond Baron and Kenny: Statistical mediation analysis in the new millennium. *Communication Monographs, 76*(4), 408-420. https://doi.org/10.1080/03637750903310360

1. Faul, F., Erdfelder, E., Lang, A.-G., & Buchner, A. (2007). G*Power 3: A flexible statistical power analysis program for the social, behavioral, and biomedical sciences. Behavior Research Methods, 39(2), 175–191. https://doi.org/10.3758

Wang, Y. A., & Rhemtulla, M. (2021). Power analysis for parameter estimation in structural equation modeling: A discussion and tutorial. *Advances in Methods and Practices in Psychological Science*, *4*(1). https://doi.org/10.1177/2515245920918253

1. Fritz, M. S., & MacKinnon, D. P. (2007). Required sample size to detect the mediated effect. *Psychological Science, 18*(3), 233-239. PMCID: PMC2843527
2. Shonkoff, J. P. (2010). Building a new biodevelopmental framework to guide the future of early childhood policy. *Child development*, *81*(1), 357-367. DOI: 10.1111/j.1467-8624.2009.01399.
3. Burgess, M., White, B., Sá, A., CQI Prevention Committee. (2024, July 31). *Amplifying Parent Voice: Utilizing the CQI Process to Garner Caregiver Feedback for HealthySteps*. Children’s Home Society of North Carolina. Presented at the ZERO TO THREE Annual Conference. Long Beach, CA.
4. Crnic KA & Booth CL (1991) Mothers’ and fathers’ perceptions of daily hassles of parenting across early childhood. *Journal of Marriage and the Family*. 53: 1043–105
5. Tamis-LeMonda, C. S., Kachergis, G., Masek, L. R., Gonzalez, S. L., Soska, K. C., Herzberg, O., Xu, M., Adolph, K. E., Gilmore, R. O., Bornstein, M. H., Casasola, M., Fausey, C. M., Frank, M. C., Goldin-Meadow, S., Gros-Louis, J., Hirsh-Pasek, K., Iverson, J., Lew-Williams, C., MacWhinney, B., Marchman, V. A., … Yurovsky, D. (2024). Comparing apples to manzanas and oranges to naranjas: A new measure of English-Spanish vocabulary for dual language learners. *Infancy : the official journal of the International Society on Infant Studies*, *29*(3), 302–326. https://doi.org/10.1111/infa.12571
